# Formulating causal questions and principled statistical answers

**DOI:** 10.1002/sim.8741

**Published:** 2020-09-23

**Authors:** Els Goetghebeur, Saskia le Cessie, Bianca De Stavola, Erica EM Moodie, Ingeborg Waernbaum, “on behalf of” the topic group Causal Inference (TG7) of the STRATOS initiative

**Affiliations:** ^1^ Department of Applied Mathematics, Computer Science and Statistics Ghent University Ghent Belgium; ^2^ Department of Medical Epidemiology and Biostatistics Karolinska Institutet Stockholm Sweden; ^3^ Department of Clinical Epidemiology/Biomedical Data Sciences Leiden University Medical Center Leiden The Netherlands; ^4^ Great Ormond Street Institute of Child Health University College London London UK; ^5^ Division of Biostatistics McGill University Montreal Quebec Canada; ^6^ Department of Statistics Uppsala University Uppsala Sweden

**Keywords:** causation, instrumental variable, inverse probability weighting, matching, potential outcomes, propensity score

## Abstract

Although review papers on causal inference methods are now available, there is a lack of introductory overviews on *what* they can render and on the guiding criteria for choosing one particular method. This tutorial gives an overview in situations where an exposure of interest is set at a chosen baseline (“point exposure”) and the target outcome arises at a later time point. We first phrase relevant causal questions and make a case for being specific about the possible exposure levels involved and the populations for which the question is relevant. Using the potential outcomes framework, we describe principled definitions of causal effects and of estimation approaches classified according to whether they invoke the no unmeasured confounding assumption (including outcome regression and propensity score‐based methods) or an instrumental variable with added assumptions. We mainly focus on continuous outcomes and causal average treatment effects. We discuss interpretation, challenges, and potential pitfalls and illustrate application using a “simulation learner,” that mimics the effect of various breastfeeding interventions on a child's later development. This involves a typical simulation component with generated exposure, covariate, and outcome data inspired by a randomized intervention study. The simulation learner further generates various (linked) exposure types with a set of possible values per observation unit, from which observed as well as potential outcome data are generated. It thus provides true values of several causal effects. R code for data generation and analysis is available on www.ofcaus.org, where SAS and Stata code for analysis is also provided.

## 1 INTRODUCTION

The literature on causal inference methods and their applications is expanding at an extraordinary rate. In the field of health research, this is fuelled by opportunities found in the rise of electronic health records and the revived aims of evidence‐based precision medicine. One wishes to learn from rich data sources how different exposure (or treatment) levels *causally* affect expected outcomes in specific population strata so as to inform treatment decisions. Neither the mere abundance of data nor the use of a more flexible model paves the road from association to causation.

Experimental studies have the great advantage that treatment assignment is randomized. A simple comparison of outcomes on different randomized arms then yields an intention‐to‐treat effect as a robust causal effect measure. However, nonexperimental or observational data remain necessary for several reasons. (1) Randomized controlled trials (RCTs) with experimental treatments tend to be conducted in rather selected populations, where the targeted effect is expected to be larger, while groups vulnerable to side effects, such as children or older patients with comorbidities, are often excluded. Informed consent procedures may also lead to restricted trial populations. (2) We may seek to learn about the effect of treatments actually received in these trials, beyond the pragmatic effect of treatment assigned. This calls for an exploration of compliance with the assignment and hence for follow‐up exposure data, that is,  nonrandomized components of treatment received. (3) In many situations (treatment) decisions need to be taken in the absence of RCT evidence. (4) A wealth of patient data is being gathered in disease registries and other electronic patient records; these often contain more variables, larger sample sizes, and greater population coverage than an RCT. These needs and opportunities push scientists to seek causal answers in observational settings with larger and less selective populations, with longer follow‐up, and with a wider range of exposures and outcome types (including quality of life and adverse events).

Statistical causal inference has made great progress over the last quarter century, deriving new estimators for well‐defined estimands using new tools such as directed acyclic graphs (DAGs) and structural models for potential outcomes.^1^, ^2^, ^3^ However, research papers—both theoretical and applied—tend to select an analysis method without formalizing a clear causal question first, and often describe published conclusions in vague causal terms missing a clear specification of the target of estimation. Typically, when this is specified, that is, there is a well‐defined estimand, a range of techniques can yield (asymptotically) unbiased answers under a specific set of assumptions. Several overview papers and tutorials have been published in this field. They are mostly focused, however, on the properties of one particular technique without addressing the topic in its generality. Yet in our experience, much confusion still exists about what exactly is being estimated, for what purpose, by which technique, and under what plausible assumptions. Here, we aim to start from the beginning, considering the most commonly defined causal estimands, the assumptions needed to interpret them meaningfully for various specifications of the exposure variable, and the levels at which we might intervene to achieve different outcomes. In this way, we offer guidance on understanding what questions can be answered using various principled estimation approaches while invoking sensibly structured assumptions.

We illustrate concepts and techniques referring to a case study exemplified by simulated data, inspired by the Promotion of Breastfeeding Intervention Trial (PROBIT),^4^ a large RCT in which mother‐infants pairs across 31 Belarusian maternity hospitals were randomized to receive either standard care or an offer to follow a breastfeeding encouragement program. Aims of the study were to investigate the effect of the program and breastfeeding on a child's later development. We generated simulated data to examine weight achieved at age 3 months as the outcome of interest in relation to a set of exposures defined starting from the intervention and several of its downstream factors. Although our motivating data stem from an RCT, the study also exemplifies questions faced in observational studies when considering downstream exposures, such as adherence to the program or starting breastfeeding. This happens because their relationship with the outcome is confounded by other variables. Our simulation goes beyond mimicking the “observed world” by also simulating for every study participant how different exposures strategies would lead to different potential responses. We call this the *simulation learner* PROBITsim and refer to the setting as the breastfeeding encouragement program (BEP) example.

Our aim here is to give practical statisticians a compact but principled and rigorous operational basis for applied causal inference for the effect of point (ie, baseline) exposures in a prospective study. We build up concepts, terminology, and notation to express the question of interest and define the targeted causal parameter. We will primarily focus on continuous outcomes where average treatment effects are of interest, although many of the concepts we discuss are valid in general. In Section 2, we lay out the steps to take when conducting this inference, referring to key elements of the data structure and various levels of possible exposure to treatment. Sections 2 also presents the potential outcomes framework with underlying assumptions and formalizes causal effects of interest. In Section 3, we describe PROBITsim, our simulation learner. We then outline various estimation approaches under the no unmeasured confounding assumption and under the instrumental variable assumption in Section 4. We explain how the approaches can be implemented for different types of exposures, and apply the methods in the simulation learner in Section 5. We end with an overview that highlights overlap and specificity of the methods as well as their performance in the context of PROBITsim, and more generally. R code for data generation, R, SAS, and STATA code for analysis, and slides that accompany this material and apply the methods to a second case study are available on www.ofcaus.org and the linked GitHub depository https://github.com/IngWae/Formulating‐causal‐questions.^5^

## 2 FROM SCIENTIFIC QUESTIONS TO CAUSAL PARAMETERS

Causal questions ask what would happen to outcome *Y*, had exposure *A* been different from what is observed. To formalize this, we will use the concept of potential outcomes^6^, ^7^ that captures the thought process of *setting* the treatment to values a∈𝒜, a set of possible treatment values, without changing any preexisting covariates or characteristics of the individual. Let $Y_{a(a)}$ be the potential outcome that would occur if the exposure were set to take the value *a*, with notation a(a) indicating the action of *setting A* to *a*. This definition is equivalent to Pearl's *do* operator, whereby the distribution *f* of Y when *A* is set to a is denoted by f(Y|do(A=a)).^1^ In what follows we will refer to *A* as either an “exposure” or a “treatment” interchangeably. Since individual‐level causal effects can never be observed, we focus on expected causal contrasts in certain populations. In the BEP example there are several linked definitions of treatment; these include “offering a BEP,” “following a BEP,” starting breastfeeding, or “following breastfeeding for 3 full months.” Each of them may require a decision of switching the treatment on or off. Ideally this decision is informed by what outcome to expect following either choice.

It is important that causal contrasts should reflect the research context. Hence in this example one could be interested in evaluating the effectiveness of the program for the total population or in certain subpopulations. However, for some subpopulations the intervention may not be suitable and thus assessing causal effects in such subpopulations would not be useful.

Consider the following question: “Does a breastfeeding intervention, such as the one implemented in the PROBIT trial, increase babies' weight at 3 months?” Despite its simplicity, empirical evaluation of this question involves its translation into meaningful quantities to be estimated. This requires several intermediate steps:

1. Define the treatment and its relevant levels/values corresponding to the scientific question of the study.
2. Define the outcome that corresponds to the scientific question(s) under study.
3. Define the population(s) of interest.
4. Formalize the potential outcomes, one for each level of the treatment that the study population could have possibly experienced.
5. Specify the target causal effect in terms of a parameter, that is, the *estimand*, as a (summary) contrast between the potential outcome distributions.
6. State the assumptions validating the causal effect estimation from the available data.
7. Estimate the target causal effect.
8. Evaluate the validity of the assumptions and perform sensitivity analyses as needed.

Explicitly formulating the decision problem one aims to solve or the hypothetical target trial one would ideally like to conduct^8^ may guide the steps outlined above. In the following we expand on steps 1‐5 before introducing the simulation learner in Section 3 and discussing steps 6‐8 in Section 4.

### 2.1 Treatments

Opinions in the causal inference literature differ on how broad the definition of “treatment” may be. Some say that the treatment should be manipulable, like giving a drug or providing a breastfeeding encouragement program.^9^ Here, we take a more liberal position which would also include for example genetic factors or even (biological) sex as treatments. Whichever the philosophy, considered levels of the treatments to be compared need a clear definition, as discussed below.^10^

Treatment definitions are by necessity driven by the context in which the study is conducted *and* the available data. The causal target may thus differ for a policy implementation or a new drug registration, for instance, or whether the data are from an RCT or administrative data. In the BEP example we may wish to define the causal effect of a breastfeeding intervention on the babies' weight at 3 months.

There are several alternative specifications of a “breastfeeding treatment” possible. Below we list a few which are interconnected and represent different types of treatment decisions:

- *A*_1_: (randomized) treatment prescription, for example, an encouragement program was offered to pregnant women.
- *A*_2_: uptake of the intervention, for example, the woman participated in the program (when offered), which may include talking to a lactation consultant, reading brochures on breastfeeding.
- *A*_3_: uptake of the target of intervention, for example, the mother started breastfeeding.
- *A*_4_: completion of the target of intervention, for example,the mother started breastfeeding and continued for 3 months.

Each of these treatment definitions *A*_*k*_,  *k* = 1, … , 4, refers to a particular breastfeeding event taking place (or not). A public health authority will be more interested in *A*_1_ because it can only decide to offer the BEP or not; an individual mother's interest will be in the effect of *A*_2_, *A*_3_, and *A*_4_ because she decides whether to participate in the program, to start, and to maintain breastfeeding. For any one, several possible causal contrasts may be of interest and are estimable. See Section 2.6.

It is worth noting that these various definitions are not all clear‐cut. For example, while *A*_4_ = 1 may be most specific in what it indicates, *A*_4_ = 0 represents a whole range of durations of breastfeeding: from “none” to “almost 3 months.” In the same vein, *A*_3_ = 1 represents a range of breastfeeding durations that follow initiation, against *A*_3_ = 0 which implies no breastfeeding at all. The variation in underlying levels of treatment could be seen as multiple versions of the treatment; we consider this topic further in Section 3.2.

**FIGURE 1:**
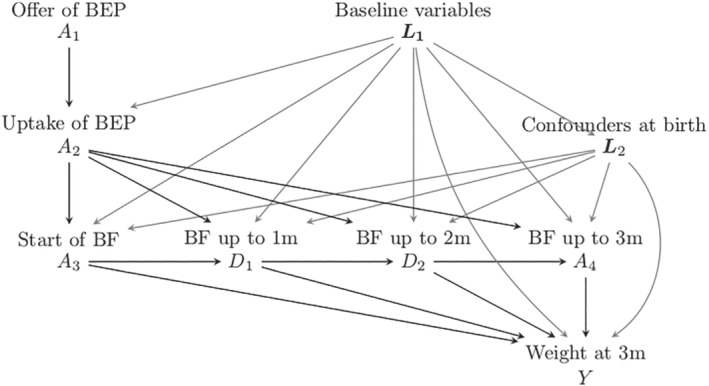
Data generating model for the simulation learner. BEP, breastfeeding encouragement program; BF, breastfeeding; m, months

Intervening at a certain stage in the “exposure chain” likely affects downstream exposure levels, as reflected in Figure 1. This is the setup we have used to generate the simulation learner data set (see Section 3), with the BEP being only available to those randomized, and where uptake of the program increases the probability of *A*_3_ = 1 and, importantly, also increases breastfeeding duration among women who initiate breastfeeding. There are of course many further aspects of the breastfeeding process that could be considered when defining exposures that are downstream from an initial randomized intervention, for example, maternal diet, the timing and frequency of breastfeeding, exclusive vs predominant breastfeeding, and so on; however for didactic purposes, we shall omit such considerations.

### 2.2 Outcomes

Similar to the definition of the treatment, it is important to carefully define the outcome *Y*. In the BEP example, the outcome of interest could be the infant's weight at 3 months, or the increment between birth weight and weight at 3 months or whether the infant is above a certain weight at 3 months. Typically, the distribution of both the absolute weight and weight gain are of interest: a BEP may well increase mean weight at 3 months by 200 g but also increase the number of overweight infants. Clarity of which outcome definition corresponds to the question of interest is therefore crucial.

### 2.3 Populations and subpopulations

A causal effect will in most cases vary across subgroups due to its dependence on baseline characteristics (effect modification). One may then be interested in the causal effects in several relevant subpopulations. It is therefore important to identify and describe the (sub)population to whom a stated effect pertains. Researchers and policy‐makers might want to study whether the breastfeeding intervention is substantially more effective for infants of less educated women who may be at highest risk of being born low weight. Alternatively they could be interested in the effect of treatment in the subpopulation of those who are actually exposed (the “treated,” as discussed above). The definition of these subpopulations involves conditioning on certain characteristics (respectively, education level and treatment received) and leads to focusing on conditional effects (see Section 4.1).

In the next section we will develop causal effects for the different subpopulations. In most settings we want to consider populations of individuals who have the possibility of receiving all treatment levels of interest. This restriction is referred to as *the positivity assumption*.^11^ It could be violated, for example, if the target population included women for whom breastfeeding is precluded (because of preexisting or pregnancy‐related conditions). Studying the effect of breastfeeding in the subpopulation of infants whose mothers cannot breastfeed (or indeed a larger population that includes this subgroup) may be impossible due to missing information—and indeed irrelevant.

### 2.4 Potential outcomes

As stated above, a potential outcome $Y_{a(a)}$ is the outcome we would observe if an exposure were *set* at a certain level *a*, where a(a) indicates the action of *setting A* to *a*. This notion needs some additional considerations linking it to the treatments and outcomes definitions given above. Specifically there are two commonly invoked assumptions that help achieve this: *no interference* and *causal consistency*.

#### 2.4.1 No interference

No interference means that the impact of treatment on the outcome of individual *i* is not altered by other individuals being exposed or not. At first sight this is likely justified in our setting: one baby's weight typically does not change because another baby is being breastfed. In resource poor or closely confined settings this could, however, be challenged. For instance, interference would happen when a child is affected by the consequences of a reduced immune system of other children who were not breastfed and hence becomes more susceptible to infectious diseases which may impact their weight at 3 months.

When the assumption of no interference is not met, the potential outcome definition becomes much more complex and involves the treatment assigned to other individuals.^12^ For example, if there were interference among infants living in the same household, the potential outcome of infant *i* would be defined not as $Y_{a(a)}$ but as $Y_{a_{i}(a),a_{i_{1}}(a^{*}),\ldots },a_{i_{K_{i}}(a^{†})}$, where infants *i*_1_ to $i_{K_{i}}$ belong to the same household as infant *i* and their breastfeeding status is set to take values (*a*^∗^, … , *a*^*†*^).

#### 2.4.2 Causal consistency

The assumption of causal consistency relates the observed outcome to the potential outcomes. Consistency (at an individual level) means that $Y_{a(a)}=Y$ when *A* = *a*, hence assuming consistency implies that the observed outcome in our data is the same as the potential outcome that would be realized in response to setting the treatment to the level of the exposure that was observed. This directly affects our interpretation of the estimated causal effect for the study population. It will also affect transportability to new settings in ways that may be hard to predict.

In practice this implies that the mode of receiving as opposed to choosing treatment level *A* = *a* per se has no impact on outcome. This may not be the case for many real‐life settings. For example “starting breastfeeding” (*A*_3_ = 1) potentially has multiple versions as some mothers who initiate breastfeeding may continue to do so for at least 3 months, while others may discontinue sooner. Also, breastfeeding may be exclusive or supplemented, breast milk may be fed at the breast or with a bottle, and so on. Hence it is to be expected that setting *A*_3_ to be 1 may translate into different durations and types of breastfeeding, and thus may not lead to the same infant weight at 3 months as when starting breastfeeding is a choice. More generally, it is typically the case that a treatment can come in many variations at some level of resolution. To achieve consistency then a more precise definition of treatment is required, so that observing or setting it is more likely to generate comparable effects. When there are multiple versions of a treatment, one should be aware that the estimated effect averages over the mix of the different versions that occur in the data. To go beyond this and evaluate the effect of different components or different mixes thereof typically demands more assumptions and adapted data analysis. For further discussion see References 13 and 14.

These observations relate to the importance of a well‐defined exposure^15^ and the need to be as precise as the data allow in our definition of treatment.^16^ Some authors have criticized the restriction imposed by this assumption (and hence by the potential outcomes approach to causal inference^10^). Being aware of the possibility of multiple versions of treatment should not deter us from pursuing the most relevant definition of treatment: instead it should lead us to greater precision and transparency in formulating the causal question and its transportability.

Note also that the assumption of consistency may be relaxed by rephrasing it at the distributional level (possibly conditional on baseline covariates), in the sense that consistency would concern, for example, the equality of the mean observed outcome of those with observed values *A* = *a* and the mean potential outcome had their treatment been set to *a*. Following this broader definition, any causal interpretation would be applicable only to settings where the *distribution* of the different versions of treatment equaled that in the analyzed sample.

### 2.5 Nested potential outcomes

The treatments considered here belong to a chain of exposures: when *A*_1_ is set, it has consequences for the “worlds” where *A*_2_, *A*_3_, and *A*_4_ act. Correspondingly, when *A*_3_ is set, *A*_1_, *A*_2_ become baseline covariates with consequences for the worlds that follow (see Figure 1). For example, in a world where a breastfeeding program is available (*A*_1_ is set to 1), starting breastfeeding (*A*_3_) may have a larger impact on weight at 3 months, because women who breastfeed having followed BEP may be more aware of the beneficial effects of breastfeeding and therefore continue breastfeeding for a longer period (see the paths from *A*_2_ to *Y* via *D*_1_, *D*_2_, and *A*_4_ in Figure 1). Although this article does not enter into the full framework of estimation for dynamic treatment strategies, we can benefit from additional definitions of potential outcomes that recognize the nested nature of the interventions.

Below we define worlds where setting *A*_2_ and *A*_3_ occurs under alternative scenarios that depend on how *A*_1_ was set (and, for *A*_3_, how *A*_1_ and/or *A*_2_ was set). These will be useful for the discussion in Section 2.6.

In the world where BEP is on offer to all (ie, when $a_{1}(1)$ is set for everyone in the population), the potential outcomes of participating or not participating in the BEP are defined as $Y_{a_{1}(1),a_{2}(1)}$ and $Y_{a_{1}(1),a_{2}(0)}$. Similarly in the world where BEP is not offered, we may consider the potential outcome of not participating in the BEP defined as $Y_{a_{1}(0),a_{2}(0)}$. In our example we assumed that the program was only available to the intervention group (ie, $Y_{a_{1}(0),a_{2}(1)}$ is not defined), and that the intervention would only affect outcome if the program was actually followed (ie, $Y_{a_{1}(1),a_{2}(0)}=Y_{a_{1}(0),a_{2}(0)}$). (In other settings it is conceivable that the mere invitation to BEP comes with advice that may have a direct impact on outcome under $a_{2}(0)$). Setting $a_{2}(1)$, here implies that *A*_1_ is set to 1; setting $a_{2}(0)$ can, in the BEP example, happen independently of how *A*_1_ is set. The corresponding potential outcomes are therefore denoted by $Y_{a_{2}(1)}(=Y_{a_{1}(1),a_{2}(1)})$ and $Y_{a_{2}(0)}(=Y_{a_{1}(1),a_{2}(0)}=Y_{a_{1}(0),a_{2}(0)}$).

Similarly, when interest is in the causal effect of *A*_3_, the potential outcomes of starting or not starting breastfeeding in the world with BEP on offer are $Y_{a_{1}(1),a_{3}(1)}$ and $Y_{a_{1}(1),a_{3}(0)}$, and in the world without BEP, they are $Y_{a_{1}(0),a_{3}(1)}$ and $Y_{a_{1}(0),a_{3}(0)}$. We deliberately omitted setting/fixing the possible $a_{2}$ level here, because we let it follow the natural course after setting $a_{1}(1)$, meaning that women may or may not choose to follow the BEP, after receiving the offer. The effect of breastfeeding in the world where the BEP is offered, may differ from the effect when the BEP is not available, as the BEP may not only affect the probability to start breastfeeding, but also the duration of breastfeeding for those who start.

One could be tempted to evaluate $Y_{a_{3}(1)}$ in the study context, using all available data and ignoring *A*_1_ and hence effectively averaging over the observed *A*_1_, where, by experimental design, for half of the individuals treatment is available and for half it is not. Such a distribution of BEP offer is, however, not a realistic future scenario, and hence this particular average effect measure is usually of no direct relevance.

The effect of breastfeeding may be even larger in the world where all women follow the program (ie, $a_{2}(1)$ is set, implying also $a_{1}(1)$ as we assume BEP cannot be followed unless it is offered). Here the potential outcomes of starting breastfeeding or not are $Y_{a_{2}(1),a_{3}(1)}$ and $Y_{a_{2}(1),a_{3}(0)}$. In the BEP example we assumed that the outcome, when not starting breastfeeding, did not depend on the offer of BEP (ie, there is no path from *A*_1_ to *Y* that does not involve *A*_3_). This means that $Y_{a_{1}(1),a_{3}(0)}$= $Y_{a_{1}(0),a_{3}(0)}$= $Y_{a_{2}(1),a_{3}(0)}$, and we can use the simplified notation $Y_{a_{3}(0)}$. Similarly we assumed that the outcome of completing 3 months of breastfeeding, $Y_{a_{4}(1)}$, was independent of the values at which *A*_1_ and *A*_2_ were set (ie, there are no paths from *A*_1_ and *A*_2_ to *Y* that do not involve *A*_4_, hence this simplified notation, knowing that *A*_3_ is per definition 1 if *A*_4_ =1). Table 2 thus lists a selection of the potential outcomes that are relevant to the BEF example.

**TABLE 1:** A selection of causal estimands for exposures *A*_1_ and *A*_2_

Estimand	Definition
Effect of program offer ($a_{1}$)	
${\text{ATE}}_{1}={\text{ATT}}^{a}$	Average treatment effect
	$E[Y_{a_{1}(1)}]-E[Y_{a_{1}(0)}]$
Effect of program uptake ($a_{2}$)	
ATE_2_	Average treatment effect
	$E[Y_{a_{2}(1)}]-E[Y_{a_{2}(0)}]$
ATT_2_	Average treatment effect among the treated^b^
	$E[(Y_{a_{2}(1)}|A_{2}=1,A_{1}=1]-E[Y_{a_{2}(0)}|A_{2}=1,A_{1}=1]$
ATNT_2_	Average treatment effect among the nontreated^b^
	$E[Y_{a_{2}(1)}|A_{2}=0,A_{1}=1]-E[Y_{a_{2}(0)}|A_{2}=0,A_{1}=1]$

^a^Intention‐to‐treat.

^b^Note that the ATT and ATNT for $a_{2}$ can only be derived from the (random) subgroup *A*_1_ = 1 since the program is only available within the randomized trial and to those assigned to it being offered.

**TABLE 2:** True average potential infant weight at 3 months under different interventions in different (sub)populations

Potential				*A*_1_ = 1	*A*_1_ = 1	*A*_1_ = 1	*A*_1_ = 0	*A*_1_ = 0	Education		
outcome	Interventions	Overall	*A*_2_ = 1	*A*_2_ = 0	*A*_3_ = 1	*A*_3_ = 0	*A*_3_ = 1	*A*_3_ = 0	Low	Int	High
$Y_{a_{1}(0)}$	BEP not offered	6017	6047	5964	6149	5733	6274	5761	5914	6057	6141
$Y_{a_{1}(1)}$	BEP offered	6115	6200	5964	6292	5733	6308	5923	6024	6155	6207
$Y_{a_{2}(0)}$	BEP not followed	6017	6047	5964	6149	5733	6274	5761	5914	6057	6141
$Y_{a_{2}(1)}$	BEP followed	6182	6200	6149	6308	5911	6329	6035	6128	6208	6226
$Y_{a_{3}(0)}$	No BF	5827	5849	5788	5871	5733	5893	5761	5730	5854	5981
$Y_{a_{1}(0),a_{3}(1)}$	BEP not offered, BF started	6214	6226	6193	6251	6133	6274	6153	6154	6248	6246
$Y_{a_{1}(1),a_{3}(1)}$	BEP offered, BF started	6249	6282	6193	6292	6157	6308	6191	6207	6276	6262
$Y_{a_{2}(1),a_{3}(1)}$	BEP followed, BF started	6277	6282	6270	6308	6212	6329	6225	6261	6292	6266
$Y_{a_{4}(1)}$	Duration BF = 3 months	6351	6345	6362	6372	6307	6392	6311	6393	6339	6286

Abbreviations: BEP, breastfeeding encouragement program; BF, breastfeeding; int: intermediate.

*A*_2_ = 1: women who followed the breastfeeding program.

*A*_2_ = 0 and *A*_1_ = 1: women who were offered the breastfeeding program but did not follow it

*A*_3_ = 1 and *A*_1_ = 1: women who started breastfeeding in the intervention group.

*A*_3_ = 1 and *A*_1_ = 0: women who started breastfeeding in the control group.

*A*_3_ = 0 and *A*_1_ = 1: women who did not start breastfeeding in the intervention group.

*A*_3_ = 0 and *A*_1_ = 0: women who did not start breastfeeding in the control group.

$Y_{a_{1}(1)}$ and $Y_{a_{1}(0)}$: the potential outcome that would occur if randomization *A*_1_ were set to take the value 1, 0, respectively.

$Y_{a_{2}(1)}$ and $Y_{a_{2}(0)}$: the potential outcome that would occur if *A*_2_ were set to 1 (which implies that *A*_1_ is set to 1) or 0. We assumed that the effect of $a_{2}(0)$ does not depend on whether BEP was available; *A*_1_ was set to 1 or 0.

$Y_{a_{3}(0)}$: the potential outcome under no breastfeeding.

$Y_{a_{1}(0),a_{3}(1)}$: The potential outcome under a double intervention with *A*_1_ set to 0 and *A*_3_ set to 1. Similar for $Y_{a_{1}(1),a_{3}(1)},Y_{a_{2}(1),a_{3}(1)}$.

$Y_{a_{4}(1)}$, the effect of completing 3 months of breastfeeding.

Results for $Y_{a_{1}(0)}$ and $Y_{a_{2}(0)}$ are equal, because BEP only affects the outcome if the program is followed.

Results for $Y_{a_{3}(0)}$ do not depend on whether *A*_1_ or *A*_2_ were set to 1 or 0 because BEP only affects *Y* via *A*_3_ and duration of breastfeeding, if started. Hence ($Y_{a_{3}(0)}=Y_{a_{1}(0),a_{3}(0)}=Y_{a_{1}(1),a_{3}(0)}=Y_{a_{2}(1),a_{3}(0)}$). The effect of full 3 months of breastfeeding is not affected by BEP.

### 2.6 Causal parameters

The next step is to contrast potential outcomes under different settings of exposure variables. We do so by defining an estimand in a well‐defined (sub)population. Individual causal effects cannot be computed since each individual can only be assigned to one treatment at a time as, via consistency, one and only one potential outcome can be observed. However, population summary measures can be estimated (under additional assumptions to be discussed below) for different groups, such as the total population or the subpopulation of treated (or untreated) individuals. Also, causal effects can be defined on different scales. In this article we focus on the mean difference as the contrast of interest.

Table 1 describes a selection of causal parameters for exposures *A*_1_ and *A*_2_. The first estimand for *A*_1_ listed in the table is the average treatment effect in the population (ATE_1_) and corresponds to the question “What would the average infant weight be at 3 months had all mothers been offered the BEP, vs the average infant weight had the mothers not been offered the program?” It is defined as ${\text{ATE}}_{1}=E[Y_{a_{1}(1)}]-E[Y_{a_{1}(0)}]$, which is equal to the intention to treat effect (ITT) of the randomized trial.

There are several possible contrasts involving uptake of the intervention *A*_2_. We could target the causal question “What would the average infant weight be at 3 months had all mothers attended the BEP, vs the average infant weight had none of the mothers attended the program?” over the whole infant population, leading to ${\text{ATE}}_{2}=E[Y_{a_{2}(1)}]-E[Y_{a_{2}(0)}]$. We might also consider this effect only within the population of women who chose to accept the offer and did attend the BEP. The latter would be the ATT. Because in our example the BEP is only available to those who are offered it, the treated population are those with *A*_2_ = 1 and *A*_1_ = 1; see Table 1. The effect in the population, ATE_2_ would be of overall interest to the developers of the BEP, as would the average treatment effect in the nontreated (ATNT_2_) because the latter would quantify the gain to be expected from a more convincing promotion campaign for the current program with larger attendance, that is, a greater *P*(*A*_2_ = 1|*A*_1_ = 1). By contrast, ATT_2_ might be of greater interests to mothers following BEP, as this would provide a measure of the expected benefit from their own uptake of the BEP offer.

Furthermore, causal effects may be heterogeneous across observable strata, for instance if the breastfeeding treatment has different causal effects depending on the education level of the mother. Thus causal effects specific to baseline subgroups would be of interest, for example, the average causal effect among those with low education could be compared with the average causal effect among those with high education. We can also define a causal effect conditional on multiple characteristics such as the expected causal effect of the program in the group of 30‐year‐old smoking mothers with a child born by caesarian section.

## 3 THE SIMULATION LEARNER

To illustrate concepts and support our learning, we generated data inspired by a real investigation but enriched by the generation of potential outcome data in addition to “observed” data. We took our inspiration from the Promotion of Breastfeeding Intervention Trial^4^ (PROBIT). PROBIT randomized mother‐infant pairs in clusters to receive either standard care or a breastfeeding encouragement intervention. Unlike the main trial, our simulation randomized individual mother‐infant pairs and focused on weight achieved at age 3 months, in a study population of babies surviving the first 3 months. Our simulation learner is therefore not a close replication of PROBIT, as we sought to highlight complexities that were not addressed in the original trial. Our aim was to discuss four (linked) definitions of treatment, for which different causal effects (ATE, ATT, etc) pertaining to corresponding treatment decisions would be of interest. This was achieved by generating realistic confounding patterns and interactions, the latter between some of the confounders and duration of breastfeeding. The confounders considered are depicted in Figure 1. In Appendix 1 (supplementary material) one can read how the mother's level of education and smoking status, just like the infant's birthweight was made to interact with breastfeeding duration to arrive at the causal effects on the expected weight at 3 months. Thus there is no direct relationship between the trial results and the causal estimates obtained from the simulated data (see Appendix 1 for more details).

### 3.1 Generating the variables

Figure 1 outlines the main relationships among the simulated variables. The baseline variables *L*_1_ were mother's age, location of living (urban vs rural and western versus eastern region), level of education (low, intermediate, high), maternal history of allergy, and smoking during pregnancy. The variables related to the infant's birth *L*_2_ were sex of child, birth weight, and birth by caesarian section. Thus, *L*_1_ are confounders of the relationship between *A*_2_ and *Y*, and (*L*_1_, *L*_2_) are confounders of the relationship between *A*_3_ and *Y*. The distribution of these variables was made to resemble that of the PROBIT study and the sample size *n* was set to 17 044, as in that study. Details of the data generation process can be found in Appendix 1 and in the material available at www.ofcaus.org; an overview is given below.

The offer of the program (*A*_1_) was assigned randomly, but the uptake of program (*A*_2_), starting breastfeeding (*A*_3_), and the duration of breastfeeding (*A*_4_) were all affected by variables at baseline (*L*_1_) or at birth (*L*_2_), with their union denoted by the vector *L*. We made the simplifying assumptions that *L*_2_ were unaffected by the program offer, that the program was only available to women in the intervention group, and that the intervention would only affect outcome if the program was actually followed. The odds of following the program after receiving an offer was assumed to depend on maternal age, education, and smoking during pregnancy, such that older and more highly educated women had a higher probability of following the program, while smokers were less likely to do so.

Following the program, that is, *A*_2_ = 1, was set to influence weight at 3 months in two ways: it increased the probability of starting breastfeeding, and increased the duration of breastfeeding if started. Older and more highly educated women and women who did not smoke during pregnancy were more likely to start breastfeeding, while having a child with lower birth weight or a baby girl decreased the probability of starting breastfeeding. The uptake of the program, higher age, higher education, not smoking, a higher birth weight, and maternal allergies were set to increase the total duration of breastfeeding, while delivery by caesarian or a having baby boy to lower it. The outcome (weight at 3 months) was set to be affected by the duration of breastfeeding and by the baseline and birth variables, some of which (smoking, education and birth weight) also modified the effect of breastfeeding.

For each woman in the simulated data set, we observed realized values of *A*_1_, *A*_2_, *A*_3_, and *A*_4_ and of the weight of the child after 3 months. In addition, several potential outcomes were generated representing the potential weight at 3 months of the child under different interventions on *A*_1_, *A*_2_, *A*_3_, and *A*_4_. This means that in our data set, for each woman the potential weight of her child at 3 months is known under different scenarios: if she had received the offer for the BEP, if she had not received the offer, if she had followed the program, if she had or had not started breastfeeding, and if she had continued breastfeeding for 3 months. Our simulations generated correlated potential outcomes, but the causal parameters introduced so far are not affected by this. We see this as an advantage since there is an intrinsic lack of information on the joint distribution of the potential outcomes in observed data. Table 2 gives the expected value of the different potential outcomes overall and in specific strata (subpopulations). These values were obtained from a very large simulated data set of five million observations and are here considered to represent the truth.

### 3.2 Different causal contrasts

From Table 2 we can derive several true causal contrasts. For example the average treatment effect (ATE) of the BEP offer is ${\text{ATE}}_{1}=E[Y_{a_{1}(1)}]-E[Y_{a_{1}(0)}]=6115-6017=98$ g. This effect may be of interest to policy makers as it is the overall mean change in infant weight at 3 months due to inviting expectant women to attend the BEP. Comparing the scenario where everyone actually receives the offer and follows the BEP with no program, the expected weight gain is ${\text{ATE}}_{2}=E[Y_{a_{2}(1)}]-E[Y_{a_{2}(0)})=165$ g. Among women who actually follow the program (the treated), the effect of BEP uptake is ${\text{ATT}}_{2}=E[Y_{a_{2}(1)}|A_{2}=1]-E[Y_{a_{2}(0)}|A_{2}=1]$ = 153 g. The effect of participating in the BEP among women who have the opportunity to follow it but opt not to, is ${\text{ATNT}}_{2}=E[Y_{a_{2}(1)}|A_{2}=0,A_{1}=1]-E[Y_{a_{2}(0)}|A_{2}=0,A_{1}=1]$ = 185 g. ATNT_2_ is larger than ATT_2_ because women who would benefit most from the BEP were, in our simulated data set, less inclined to follow it.

In this tutorial, we are treating *A*_1_, *A*_2_, *A*_3_, and *A*_4_ as point exposures, that is, as exposures to be examined separately, with any previous exposures in the chain treated as background variables. In other words, for each targeted treatment, we consider the time point at which it is implemented. We then ask about the impact of setting this treatment to a given value, conditional on background information. In the setting of our study: when *A*_3_, the decision to start breastfeeding is implemented, the values of *A*_1_ and *A*_2_ are already known and the baby has been born. The set of information carried by *A*_1_ and *A*_2_ could be treated as baseline information, like *L*, conditional on which the effect of starting breastfeeding is measured.

Alternatively, we could consider the joint impact of multiple interventions. Using the nested potential outcomes notation introduced in Section 2.5, we could address the question “What would the average infant weight at 3 months be, had all mothers started breastfeeding vs the average infant weight had they not started at all?” under different worlds where *A*_1_ and *A*_2_ are set to take different values. In the world without BEP, the answer would be ${\text{ATE}}_{3,a_{1}(0)}=E[Y_{a_{1}(0),a_{3}(1)}]-E[Y_{a_{1}(0),a_{3}(0)}]$ = 387 g. In the world where the BEP is offered, the gain in weight at 3 months would be substantially higher: ${\text{ATE}}_{3,a_{1}(1)}=E[Y_{a_{1}(1),a_{3}(1)}]-E[Y_{a_{1}(1),a_{3}(0)}]=422$ g. The weight gain in the world where everyone followed the program would be ${\text{ATE}}_{3,a_{2}(1)}=Y_{a_{2}(1),a_{3}(1)}-Y_{a_{2}(1),a_{3}(0)}=450$ g. This is the largest effect because, in the simulation, BEP increases the mean duration of breastfeeding. In general there are greater average potential outcomes with increased intensity of the joint interventions.

The average treatment effect in the treated (with respect to *A*_3_) also differs between randomization worlds because more women among those randomized to receive the BEP will start breastfeeding than in the control group. The effect of breastfeeding in those who started breastfeeding and are in the intervention arm (ie, *A*_1_ = 1) is equal to ${\text{ATT}}_{3,a_{1}(1)}=E[Y_{a_{1}(1),a_{3}(1)}|A_{3}=1,A_{1}=1]-E[Y_{a_{1}(1),a_{3}(0)}|A_{3}=1,A_{1}=1]=421$ g, and the effect of breastfeeding in those who started breastfeeding but are in the control arm (ie, *A*_1_ = 0) is ${\text{ATT}}_{3,a_{1}(0)}=381$ g. The average effect of breastfeeding in those who did not start breastfeeding is ${\text{ATNT}}_{3,a_{1}(1)}=424$ g when the program is available and ${\text{ATNT}}_{3,a_{1}(0)}=393$ g when not.

We could also ask the question “What would the average infant weight at 3 months be, had all mothers breastfeed for 3 months vs the average infant weight had they not started at all?” As noted before, setting *A*_4_ = 0 will include a very heterogeneous set of breastfeeding behaviors, as well as not breastfeeding at all. A more refined question would restrict the comparison to a setting where there is no breastfeeding at all, that is, $E[Y_{a_{4}(1)}]-E[Y_{a_{3}(0)}]=6351-5827=524$ g.

When implementing an intervention, it is of interest to identify those subgroups for which the intervention is most beneficial. Table 2, for example, shows that the infants of mothers in the lowest stratum of education would gain more than those of mothers in the highest, both when the intervention is offering the program $E[Y_{a_{1}(1)}|L=\text{low}]-E[Y_{a_{1}(0)}|L=\text{low}]=110$ g and when the intervention is following the program $E[Y_{a_{2}(1)}|L=\text{low}]-E[Y_{a_{1}(0)}|L=\text{low}]=214$ g, as opposed to 66 and 85 g for women in the highest stratum of education.

Some of the causal effects described above are not realistic. For example, the largest causal contrast is the expected weight gain when every infant is breastfed for the full 3 months vs the expected weight gain when no one is breastfed (524 g above). However not all women can or wish to start breastfeeding (nor would all women willingly refrain from it). As alluded to in the discussion of positivity in Section 2.3, a woman who is very ill at the end of pregnancy may not have the option of breastfeeding her baby because of toxicity of prescribed medication or ill‐health. It follows that considering the intervention where every woman continues breastfeeding for the full 3 months is even less realistic. It is important to define the causal question precisely in a pertinent population before turning to estimation.

## 4 PRINCIPLED ESTIMATION APPROACHES

The estimation approaches discussed here rely on further assumptions in addition to those outlined in Section 2.4. These can be classified according to whether or not they invoke the *no unmeasured confounding* (NUC) assumption which states that the received treatment is independent of the potential outcomes, given covariates ***L***. Formally, the NUC assumption states: $(Y_{a(0)})⊥A|L$ and $(Y_{a(1)})⊥A|L$, where, hereafter *A* denotes a binary exposure. In other words, the assumptions states that a sufficient set of variables ***L*** that confound the exposure/outcome relationship have been measured and are available to the analyst.

The estimation approaches that rely on the NUC assumption include standard outcome regression and propensity score (PS) based methods such as PS stratification, regression adjustment, matching, and inverse probability weighting. These are reviewed below. Alternatively, if an instrumental variable (IV) is available, IV methods can be used by also invoking additional assumptions in place of NUC. IV definitions an assumptions are described in Section 4.2.

### 4.1 Methods based on the no unmeasured confounders assumption

When a sufficient set of confounders ***L*** is measured, the causal effect of treatment can be estimated by comparing observed outcomes between the treated and untreated people with identical values for ***L***. Such direct control for ***L*** may be done in different ways: by regression or stratification or matching. We discuss these approaches in the next subsections.

#### 4.1.1 Initial data summary and the propensity score

Before proceeding with the analysis one should examine how treatment groups differ in their population mix—that is, examine the imbalance in covariates between treatment groups as exemplified in Appendix 2 (supplementary material). The existence of substantial residual imbalance could lead to residual confounding in the effect estimate and may call for a sensitivity analysis.

**FIGURE 2:**
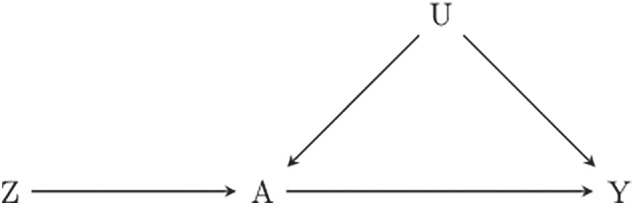
DAG representing the setting for an IV analysis. *A*, treatment; *U*, unmeasured confounders; *Y*, outcome; *Z*, instrument

When *L* includes only few variables, this balance check can be achieved visually (eg, using balancing plots as in Appendix 2, Figures 2, 5, and 6) or by reporting mean or percentage differences between treatment groups for each variable, as in Appendix 2, Table 1. With high‐dimensional *L* this information is preferably summarized through the *propensity score*. The propensity score (PS) is the probability of being treated conditional on the covariates, *e*(***L***) = *P*(*A* = 1|***L***).^17^ The PS is an important function of the covariates that reduces the (possibly high‐dimensional) vector ***L*** into a scalar containing all measured information that is relevant for the treatment assignment in relation to the outcome. This property score enjoys the so‐called balancing property, meaning that the covariate distributions of the treated and nontreated are exchangeable (the same) when conditioning on the PS. Intuitively, the role of the PS can be thought of as one of restoring balance between treated and untreated groups once conditioned upon. For example, if we were to compare all treated subjects with untreated subjects who all had the same value of the PS, the distribution of the covariates ***L*** would be the same, much like in a randomized trial. However unlike in a randomized trial, balance is not achieved between the treated and untreated groups for any covariates that were not included in the PS. The balancing property implies that all relevant confounding information in ***L*** is contained in *e*(***L***), so that if $(Y_{a(0)},Y_{a(1)})⊥A|L$, then also $\left(Y_{a(0)},Y_{a(1)})⊥A|e(L\right)$. This implies that *e*(***L***) can be used instead of the full vector ***L***.

The PS is estimated from the data, usually by fitting a parametric (eg, logistic regression) model for the probability of being treated given the confounding variables, although a variety of other approaches can be employed including tree‐based classification.^18^ However derived, the adequacy of the estimated PS, $\hat{e}(L)$, as a balancing summary of the confounder distributions across treatment groups must be evaluated^19^ by checking whether $L⫫A|\hat{e}(L)$). While balance of the joint distribution of the confounders ***L*** is required, in practice balance is often assessed for each confounder *L* ∈ ***L*** separately by comparing standardized mean differences, variance ratios, and other distributional statistics and plots such as empirical cumulative distribution plots, between the treated and untreated groups after weighting, stratification, or matching by the estimated PS.^20^ We illustrate some of these checks in Appendix 2. To date, variable selection for PS modeling is done largely on a trial and error basis, beginning with a model thought to contain all relevant confounders and adding higher order terms (polynomials, interactions) if balance appears not to have been achieved.^21^

The PS can also be used to examine the positivity assumption by checking for overlap of the propensity score distribution of those who are treated and those who are not. For this reason, automatic variable selection approaches (eg, stepwise) or prediction‐based measures of fit (eg, C‐statistic), which seek best prediction of treatment allocation when specifying the PS model, may not provide the best balance for the confounders and favor variables that are strongly predictive of the treatment, even if they are only weakly or not at all predictive of the outcome.^22^

#### 4.1.2 Outcome regression

Perhaps the simplest and most familiar form of causal estimation is outcome regression. In this approach, a model is posited for the outcome as a function of the exposure and the covariates. For example, for a continuous outcome the linear regression model of the form

(1) $$E[Y|A,L]=\beta_{0}+\beta_{A}A+\gamma^{\prime }f(L,A),$$

where γ is a vector of parameters and *f*(***L***, *A*) is a (vector) function of ***L*** and *A* representing, for example, the main effect of the covariates ***L*** and interactions between covariates and *A*. Ordinary least squares can be used to estimate the parameters of the outcome linear regression model. The absence of any interactions between *A* and ***L*** yields

(2) $$E[Y|A,L]=\beta_{0}+\beta_{A}A.$$

Assuming no interference, consistency, and NUC, $\beta_{A}$ in (2) is interpreted as the average causal effect of *A*, that is, ATE = *A*. In the presence of interactions $\beta_{A}$ in (1) is the causal effect of *A* in the reference category of ***L***, that is, where ***L*** = **0** if *f*(***L***, *A*) = 0 occurs whenever ***L*** = **0**.

When a correct specification of the model is

$$E[Y|A,L]=\beta_{0}+\beta_{A}A+\beta_{L}^{\prime }L+\beta_{LA}^{\prime }LA,$$

$\beta_{A}+\beta_{LA}^{\prime }L$ is interpreted as the causal effect of *A* (level 1 vs 0) in the stratum defined by ***L***, hence representing conditional causal effects: the *L* stratum‐specific ATE_*L*_.

To estimate causal parameters such as those shown in Table 1, the additional step of marginalizing ATE_*L*_ over the distribution of ***L*** is needed,.

We identify the average ATE for *A* then as follows:

$$\begin{array}{ll} \text{ATE} & =E\{E[Y_{a(1)}|L]\}-E\{E[Y_{a(0)}|L]\}\;\\ & \overset{\left(2\right)}{=}E\{E[Y_{a(1)}|A=1,L]\}-E\{E[Y_{a(0)}|A=0,L]\}\;\\ & \overset{\left(3\right)}{=}E\{E[Y|A=1,L]\}-E\{E[Y|A=0,L]\}\;\\ & \overset{\left(4\right)}{=}(\beta_{0}+\beta_{A}+\beta_{L}^{\prime }E[L]+\beta_{LA}^{\prime }E[L])-(\beta_{0}+\beta_{L}^{\prime }E[L])\;\\ & =\beta_{A}+\beta_{LA}^{\prime }E[L],\; \end{array}$$

where equality (2) follows from the NUC assumption, (3) from the consistency assumption, and (4) from the assumption of correct specification of the outcome model.

These estimands can be estimated by ${\hat{\beta }}_{A}+{\hat{\beta }}_{LA}^{\prime }n^{-1}\sum_{i=1}^{n}(l_{i}),$ where *n* is the sample size. When there are no treatment‐covariate interactions (ie, $\beta_{LA}$ is a vector of zeroes), then the ATE equals $\beta_{A}$ and its standard error can be taken directly from the fitted model that does not include any interactions. Otherwise, a standard error accounting for the correlation between $\beta_{A}$ and $\beta_{LA}$ as well as estimation of *E*[***L***] must be computed either analytically or via a bootstrap procedure.

A similar approach can be taken to estimate the ATT (or the ATNT). The ATT, for instance, can be computed noting that $\text{ATT}=E\{E[Y_{a(1)}|A=1,L]\}-E\{E[Y_{a(0)}|A=1,L]$. Letting ${ℐ}_{A=1}$ denote the indices *i* of those exposed subjects and $\#{ℐ}_{A=1}=\sum_{i=1}^{n}a_{i}$ denote the number of exposed individuals (the cardinality of ${ℐ}_{A=1}$), the ATT can be estimated using the outcome regression coefficient estimates by

$$\hat{\text{ATT}}={\left(\#{ℐ}_{A=1}\right)}^{-1}\underset{i\in {ℐ}_{A=1}}{\sum }({\hat{\beta }}_{A}+{\hat{\beta }}_{LA}^{\prime }l_{i}).$$

For binary and other categorical outcomes other appropriate outcome models can be used such as the logistic regression model. This model will yield fitted values of *E*[*Y*|*A* = 1, ***L***] and *E*[*Y*|*A* = 1, ***L***] for all individuals which can then be averaged over the appropriate population.

Concerns about model misspecification may be reduced by using a more flexible model for the outcome. For example, we may consider transformations of ***L*** such as splines to specify *f*(***L***, *A*), leading to a less parametric model which, however, requires estimation of a greater number of parameters. An additional concern is the possibility that a chosen outcome model leads to extrapolations outside of the data cloud (in other words, to lack of positivity). Users should therefore be aware of this and adopt methods discussed above to assess whether lack of positivity is an issue.

When an appropriate propensity score has been estimated such that it provides the desired balance, outcome regression can also be performed with the generic function *f*(***L***, *A*) being replaced by $\hat{e}(L)$, assuming no interactions between ***L*** and *A*:

$$E[Y|A,L]=\beta_{0}+\beta_{A}A+\beta_{e(L)}\hat{e}(L).$$

This approach is known simply as *propensity score regression* with the ATE and ATT then estimated via standard regression followed by averaging over the PS as opposed to ***L***, much as in Section 4.1.2. It can be shown that for the linear outcome model the propensity score regression estimators for the ATE and ATT are consistent under correct specification of the PS, even if the outcome model is misspecified, provided the treatment effect is constant across *e*(***L***).^23^ The assumptions for propensity score regression are certainly restrictive and Table 5 provides an example of the resulting bias when they are violated.

#### 4.1.3 Stratification and PS matching

Stratification can be used to estimate the ATE by taking the weighted sum of the treatment group differences in sample means across strata defined by a combination of the covariates *L*. This is naturally only feasible if *L* is low dimensional. For example, for two binary *L*s, we could create four strata and estimate stratum‐specific ATEs and then average them using the relative frequencies of the strata. For high‐dimensional *L*, strata may be defined by categories of the propensity score (fifths—that is, using quintiles—is a common choice,^24^ but for large sample sizes increasing the number of strata will reduce the residual bias within strata). Finally, let ${\hat{\mu }}_{aj}$ denote the sample average of *Y* for those with treatment level *a* in the *j*th stratum. Then the stratification‐based estimator of the ATE is given by

$$\overset{J}{\underset{j=1}{\sum }}\left(\frac{n_{j}}{n}\right)[{\hat{\mu }}_{1j}-{\hat{\mu }}_{0j}].$$

This approach when based on the PS, will work if there is reasonable balance of values of confounders in each of the defined strata. If not, one can regress the outcome on confounders within strata and use the stratum‐specific mean predicted value instead..^25^ Standard errors for stratification‐based estimators often rely on simplifying assumptions; again, bootstrap may be used as an alternative. The ATT (and ATNT) can similarly be estimated by replacing the ratio *n*_*t*_/*n* with a ratio of the stratum proportion of the treated (untreated) population.

Matching is similar in spirit to stratification, but taken to the finest strata: the individual level. For each individual *i* in the sample, we select *M* ≥ 1 individuals, *i′*, who are matched to *i* based on some matching criterion and matching method. Then the estimators of the ATE and ATT are, respectively,

$$n^{-1}\overset{n}{\underset{i=1}{\sum }}(2A_{i}-1)\left(Y_{i}-M^{-1}\underset{i^{\prime }}{\sum }Y_{i^{\prime }}\right)\;\text{and}\;{\left(\#{ℐ}_{A=1}\right)}^{-1}\underset{i\in {ℐ}_{A=1}}{\sum }\left(Y_{i}-M^{-1}\underset{i^{\prime }}{\sum }Y_{i^{\prime }}\right),$$

where *i′* runs over the set ${ℳ}_{i}$ of individuals matched to *i*.

In practice, the following algorithm should be followed:

1. Choose a matching criterion, *C*_*i*, *i′*_ such as nearest neighbor, the Mahalanobis distance, or vector norm, and implement a matching method given the criterion. The criterion may be applied to ***L*** or to a summary such as the PS, $\hat{e}(L)$.
2. Evaluate the quality of the matched sample by carrying out balancing checks described above.
3. If balance is not satisfactory, return to step 1.

There are several factors to consider in a matched analysis, such as the number of matches per individual, *M*; whether to match with or without replacement; if matching without replacement, whether to use greedy matching or the more computationally intensive optimal matching. A discussion of the relative merits and the impact of these choices on bias and variance can be found in a review by Stuart.^26^ If balance remains unsatisfactory or to increase robustness, outcome regression as described in Section 4.1.2 can be performed within the matched sample.

Several standard softwares include packages that implement matching and, in some cases, covariate balance checks. Note that the bootstrap should not be used to compute standard errors following matching, and that suitable standard errors depend on how the matching was carried out (eg, whether with replacement or not).^27^

#### 4.1.4 Inverse probability weighting

The idea behind inverse probability weighting (IPW) is to construct a pseudosample in which there are no imbalances on measured covariates between the treatment groups. While IPW can be used for treatments measured only at baseline, its strength is with time‐varying treatments. Let *W*_*i*_ be the inverse of the probability of the *received* treatment, defined as *W*_*i*_ = *P*(*A*_*i*_ = *a*_*i*_|*L*_*i*_)^−1^ = *e*(***L***)^−1^. Assuming no interference, consistency, NUC, and correct specification of the PS model, the average potential outcome if the whole population were treated can, under causal consistency, be shown to equal

(3) $$E\left[Y(1)\right]=E\left[W_{i}A_{i}Y_{i}\right].$$

That is, the sample weighted average can be used to estimate $E[Y_{a}]$ for any a, a *marginal* mean that averages over the population distribution of covariates ***L*** 1. An alternative definition of the weights, denoted stabilized weight, is *W*_*i*_ = *P*(*A*_*i*_ = *a*_*i*_)*P*(*A*_*i*_ = *a*_*i*_|***L***_*i*_)^−1^ and is often preferred as it follows naturally from the theoretical derivation of IPW estimators^28^ and for time‐varying exposures typically leads to less extreme values and more stable estimates.^2^ In practice, an estimated PS is used in place of *P*(*A*_*i*_ = 1|***L***) and *P*(*A*_*i*_ = 1) is replaced by a simple sample average before an empirical average is taken:

(4) $$\hat{E}[Y_{a(1)}]=n^{-1}\overset{n}{\underset{i=1}{\sum }}w_{i}a_{i}y_{i},$$

where *w*_*i*_ are such estimates of *W*_*i*_. If there are *many* people with a given set of characteristics ***l***_*i*_ who are treated, but few with this characteristic who are not treated, then *P*(*A*_*i*_ = 1|***L***_*i*_ = ***l***_*i*_) will be “large” and its inverse “small” so these treated individuals will be downweighted in the sample.

Similarly, an estimate of the average potential outcome if the whole population were set to be *un*treated is

(5) $$\hat{E}[Y_{a(0)}]=n^{-1}\overset{n}{\underset{i=1}{\sum }}w_{i}(1-a_{i})y_{i}.$$

As before, if there are *many* people with a given set of characteristics who are treated, but few who are not treated, then *P*(*A*_*i*_ = 0|*L* = *l*_*i*_) will be “small” and its inverse “large” so that these people are upweighted. This approach is well‐known in the survey sampling literature,^29^ where it is used to adjust for unequal sampling fractions—typically the oversampling of certain smaller but important subgroups in a population. When the weights are extreme, they may be truncated or normalized.^30^

As before, the PS is usually estimated via a parametric model. So, similarly to previously described estimation steps, the IPW estimation procedure is straightforward and consists of:

1. Fitting the PS model, for example, logistic regression model for the probability of being treated given ***L***.
2. Calculating the weights:
  (a) Use the fitted PS to predict the probability that a person received the treatment s/he did in fact receive.
  (b) Set each individual's weight to one over the probability computed in (2a). “Stabilize” this weight by including the simple probability of being treated with the observed treatment in the numerator.
  (c) Check the confounders' balance in the weighted sample. If balance is inadequate, return to step 1 and improve the PS model specification by involving the unbalanced confounders.
3. Fitting the outcome model: weighting each individual by the weights computed in (2b), fit a regression model for the outcome given the treatment. The treatment coefficient is an estimate of the ATE.

Following the estimation procedure above, standard errors must be computed analytically or via bootstrap to account for estimation of the weights. Robust or empirical standard errors provide reasonable coverage, although they do not explicitly account for the fitting of the PS model.

To estimate the ATT, rather than the ATE, we change our focus to $E[Y_{a(1)}-Y_{a(0)}|A=1]$. Clearly, we can compute an estimate of $E[Y_{a(1)}|A=1]$ with little trouble, as this is easily identified and estimated in the data by

$$\hat{E}[Y_{a(1)}|A=1]={\left(\#{ℐ}_{A=1}\right)}^{-1}\underset{i\in {ℐ}_{A=1}}{\sum }a_{i}y_{i}.$$

The second term, $E[Y_{a(0)}|A=1]$, requires a bit more work: this is an average of the potential outcome $Y_{i,a(0)}$ in the (impossible) situation where the *i* indexes those who were in fact treated. It turns out that we can again use reweighting of the observed sample of the untreated individuals by

(6) $$\hat{E}[Y_{a(0)}|A=1]]=n^{-1}\overset{n}{\underset{i=1}{\sum }}w_{i}^{ATT}(1-a_{i})y_{i},$$

with stabilized weights equal to

$$w_{i}^{ATT}=\frac{\hat{P}(A_{i}=1|L_{i})}{\hat{P}(A_{i}=0|L_{i})}\times \frac{\hat{P}(A_{i}=0)}{\hat{P}(A_{i}=1)}.$$

As before, the weighting has been used to construct a pseudopopulation in which there are no imbalances on measured covariates between the exposure groups. In the case of the ATT, we do so by rebalancing the distribution of the covariates in the unexposed group only.

Care must be taken as, in practice, a small number of large weights can be highly influential, though this may be mitigated through ad hoc but effective solutions such as shrinking of the largest weights to a smaller value such as the 99th percentile of the weight distribution (often referred to as truncation or sometimes called “capping”).

#### 4.1.5 A hybrid approach: Doubly robust estimation

Outcome regression requires correct specification of the outcome model while the inverse propensity score weighting requires correct specification of the propensity model. The methods can be combined by *augmenting* the inverse probability of treatment weighted estimators. Note that

$$E[Y_{a(a)}]=E[Y_{a(a)}-\mu_{a(a)}(L)]+E[\mu_{a(a)}(L)],$$

where here, $\mu_{a(a)}(L)$ is the expected outcome with *A* set to *a* and covariates taking values ***L***. Invoking the consistency and NUC assumptions, we have $\mu_{a(a)}(L)=E[Y|A=a,L=l]$ which is, in practice, replaced by a parametric model. This gives rise to the alternative estimator

(7) $$\hat{E}[Y_{a(a)}]=\frac{1}{n}\overset{n}{\underset{i=1}{\sum }}\frac{I[A_{i}=a](y_{i}-\hat{\mu }(a(a),l_{i}))}{\hat{P}(A_{i}=a|L=l_{i})}+\frac{1}{n}\overset{n}{\underset{i=1}{\sum }}\hat{\mu }(a(a),l_{i}),$$

with *I*[*x*] the indicator function that takes value 1 when condition *x* holds and 0 otherwise, and ${\hat{\mu }}_{a}(l)$ a model‐predicted mean for *Y* with *A* set to *a* and ***L*** as observed. The estimator (7) is *doubly robust*, which means that it is consistent even if one of *P*(*A*_*i*_ = *a*|***L***=***l***_*i*_) and the modeled mean $\mu_{a(a)}(L)$ is misspecified. If both models are correctly specified, then the augmented inverse weighted estimator is at least as efficient as the unaugmented inverse weighted estimator.

Bang and Robins,^31^ building on Scharfstein et al,^32^ reformulated the augmented estimator, noting that it can be viewed as an unweighted regression that includes the inverse of the PS as a covariate. It appears that unlike for the PS model, one can use separate regularized regressions for the outcome and propensity score models to derive a doubly robust “g‐estimator” with standard confidence intervals that are correct given the variable selection procedure (see, eg, References 33, 34, 35, 36). The bias otherwise induced by shrinkage of the coefficients in penalized regression models is counteracted by propensity‐based adjustments with doubly robust estimation.

### 4.2 Instrumental variable based methods

All methods described so far yield valid estimates under the NUC assumption. This assumption is easily violated in observational studies, where the prognosis of patients tends to determine the choice of treatment and the reasons for a specific treatment choice are seldom completely registered or, more generally, the exposure level and outcome are influenced by unmeasured factors. One alternative approach is an instrumental variable (IV) analysis which can handle both measured and unmeasured confounding. Asymptotically unbiased estimation results once a “pseudo‐random variable” or so called “instrumental variable” is identified *and* some additional assumptions hold. The method originates from econometrics^37^, ^38^, with extensions such as generalized difference in difference (DiD) methods and control function models^39^, ^40^, ^41^ These methods are also becoming increasingly popular in medical research. The literature on IV, with examples, is vast.^42^, ^43^, ^44^, ^45^, ^46^ We will discuss here the general IV assumptions, typical causal estimands, and the corresponding estimation procedures that are most commonly used. To focus on the principles here, our formalization below ignores measured baseline covariates (which we have been denoting ***L***), although the approach extends quite naturally to conditioning on them. The unmeasured confounder(s) are denoted here by *U*.

An IV analysis aims to resemble that of a RCT, by using one or more variables (instruments) associated with treatment, but not in any other way related to the outcome. The instrument can be seen as a surrogate for randomization. This is depicted in Figure 2 where *Z*, the instrumental variable, is associated with *A* (the figure suggests a causal relation but that is not necessary, association is sufficient). The instrument *Z* is related to response *Y* only via the treatment *A*; and the instrument is independent of unmeasured confounders *U*.

Instrumental variable analysis can be used in trials to study the effect of noncompliance,^37^, ^47^, ^48^ as in our BEP example, where randomization to the offer of the breastfeeding program could be used as instrument for attending the program. Variation in preference for a certain treatment among physicians^49^, ^50^ or variation in treatment policies among medical centers^51^ are other examples of variables which can be considered close to pseudorandomization for treatment or policy assignment. When physicians have strong preferences for one or another treatment, identical patients may receive different treatments; a variable measuring the physician's preference, like the percentage of prescriptions *A* = 1 in a certain time window, could be used here as an instrument. Another popular IV approach is found in so‐called “Mendelian randomization” studies where genetic variation takes the role of the instrumental variable.^52^, ^53^

#### 4.2.1 The three core IV assumptions

To be an instrumental variable for the causal effect of *A* on *Y*, *Z* should satisfy the following three core assumptions (possibly conditional on ***L***):

IV1 *Z* is associated with the treatment *A* of interest;
IV2 *Z* is independent of any unmeasured confounders of the *A* → *Y* relationship;
IV3 *Z* is independent of the outcome *Y* conditional on treatment *A* and unmeasured confounders *U*.

Unfortunately only assumption IV1 can be empirically checked in the data.^54^ Assumptions IV2 and IV3 are not verifiable in the data: only their plausibility can be examined. For example, the observation that *Z* is independent of all observed confounders makes assumption IV2 more plausible. Situations in which these assumptions are likely or unlikely to hold are discussed for Mendelian randomization and for physician's preference by several authors.^42^, ^52^, ^53^ When *Z* is an IV and the assumptions of no interference, consistency and positivity hold, IV‐based estimation does not require the NUC assumption to lead to an estimator. However, an IV estimator on its own can only provide bounds for causal treatment effect.^55^, ^56^ These bounds are generally so wide that they are not useful. In order to obtain point estimates, additional assumptions are needed as discussed below.

#### 4.2.2 Additional assumptions to obtain an effect estimate

As the three main IV assumptions alone are not sufficient to identify causal effects, additional assumptions are needed for estimation. Often some form of homogeneity of treatment is assumed. The traditional approach, popular in econometrics is to use structural equation models which assume a constant effect of treatment across individuals. An example of a standard linear structural equation model is

$$Y=\beta_{0}+\beta_{A}A+f(U,\epsilon ),$$

with *U* the unmeasured confounder(s) and ϵ an independent error term.^57^ The parameter $\beta_{A}$ is, under the consistency assumption, equal to both the ATE and the ATT. For a binary instrument, under the three core IV assumptions, it can easily be shown that $\beta_{A}$ equals the following IV estimand:

(8) $$\frac{E[Y|Z=1]-E[Y|Z=0]}{E[A|Z=1]-E[A|Z=0]}.$$

Assuming the same treatment effect for all individuals is, in general, unrealistic. More severely affected patients may benefit more (or less) from treatment, treatment could interact with other drugs, or men and women could respond differently, for example. Assumptions regarding a homogeneous treatment effect can be relaxed by using *structural mean models* (SMM).^58^, ^59^ An SMM is a model for the mean difference between an observed outcome *Y* and a potential outcome such as $Y_{a(0)},$ that may condition on observed treatment *A* and instrument *Z*. A simple SMM is:

(9) $$E[Y-Y_{a(0)}|A,Z]=A\beta_{A}.$$

In this SMM, the homogeneity assumption is less strong and only requires that $E[Y-Y_{a(0)}|A,Z]$ does not depend on *Z*. For *A* = 0 we obtain $E[Y-Y_{a(0)}|A=0,Z]=0$, which is exactly the (mean) consistency assumption for a(0). For *A* = 1 we obtain $E[Y-Y_{a(0)}|A=1,Z]=\beta_{A}$. Since $E[Y|A=1,Z]=E[Y_{a(1)}|A=1,Z]$ because of the consistency assumption, the parameter $\beta_{A}$ in this SMM equals the ATT. Robins^58^ showed that $\beta_{A}$ in this model is exactly equal to the IV estimand (8). Baseline covariates ***L*** can be added to this model, including interactions between ***L*** and exposures *A*.^47^ Other homogeneity assumptions for IV estimation are possible, see Reference 43 for an overview.

An alternative assumption is *monotonicity* of the effect of *Z* on *A*. We discuss monotonicity briefly for a binary instrument *Z* that causally affects a binary treatment *A*. Defining $A_{z(z)}$ as the value of *A* when *Z* is set to *z* ∈ {0, 1}, four types of individuals can be identified: (1) always takers: those with $A_{z(1)}=A_{z(0)}=1$, that is, individuals who will take the treatment regardless of the value of the instrument; (2) never takers: those with $A_{z(1)}=A_{z(0)}=0$; (3) compliers: those with $A_{z(1)}=1$ and $A_{z(0)}=0$; and (4) defiers: those with $A_{z(1)}=0$ and $A_{z(0)}=1$.

The monotonicity assumption states that $A_{z(1)}\geq A_{z(0)}$, which implies that defiers do not exist. Under this assumption the IV estimand (8) identifies a “local” causal effect in the subgroup of compliers, which is the complier average causal effect (CACE):^37^, ^38^

$$CACE=E[Y_{a(1)}-Y_{a(0)}|A_{z(1)}=1\;\text{and}\;A_{z(0)}=0)].$$

The interpretation of the CACE is often difficult,^50^, ^60^, ^61^ because the subgroup of compliers cannot be identified from the data, although general characteristics like the distribution of age and sex can be obtained.^62^ In some particular instances, however, it could be the parameter of interest: the CACE represents the intervention effect in the subgroup of individuals for which it is acceptable and accepted, for example, the CACE for *A*_2_ is the effect among those individuals who will attend the breastfeeding program when invited but not otherwise. Although this formulation is appealing, defining monotonicity is more complicated when the instrumental variable is continuous, and the interpretation is often even less intuitive.^61^, ^63^

The above section shows that the interpretation of an IV analysis depends on the choice of the additional assumptions, under homogeneity assumptions the ATE or ATT is the estimand being targeted, while under monotonicity assumptions the CACE is the target estimand.

#### 4.2.3 Standard IV estimation

There are several ways of obtaining point estimates in an IV analysis. The traditional IV estimator is the Wald estimator^64^ which equals:

$${\hat{\beta }}_{IV}=\frac{\hat{cov}(Y,Z)}{\hat{cov}(A,Z)}.$$

This estimator is based on two relationships which are unconfounded: the relationship between instrument *Z* and outcome *Y*, and the relationship between instrument *Z* and treatment *A*. In case of a binary instrument, this expression reduces to

(10) $${\hat{\beta }}_{IV}=\frac{\hat{E}[Y|Z=1]-\hat{E}[Y|Z=0]}{\hat{E}[A=1|Z=1]-\hat{E}[A=1|Z=0]},$$

which is the IV estimand (8) with expectations replaced by simple averages; $\hat{E}[Y|Z=z]$ refers to the average of *Y* in the selected subset with *Z* = *z* ∈ {0, 1}. Similarly, $\hat{E}[A|Z=z]$ is a simple average of *A* in the selected subset with *Z* = *z*. The numerator of (10) expresses the effect of the instrument on the outcome; the mean difference in outcome between those with *Z* = 1 and *Z* = 0, or the risk difference in the case of a binary outcome. To obtain an estimate of the treatment effect on the outcome, the effect of the instrument on the outcome is inflated by dividing the numerator by the effect of the instrument on the treatment. The smaller the correlation between *Z* and *A* (the so‐called strength of the instrument), the larger the inflation factor.

The traditional IV estimator (10) can be equivalently obtained through a two stage linear regression (2SLS) approach. In the first stage, a linear (OLS) regression model is fitted with treatment *A* as dependent variable and the instrument *Z* as an independent variable (and optionally measured confounders *L*), yielding for each subject $\hat{E}[A|Z=z_{i}]$. In the second stage, a linear regression model is fitted to the outcome *Y* on $\hat{E}[A|Z]$ (and possibly *L* ^65^). The regression coefficient for $\hat{E}[A|Z]$ is the IV estimator of the treatment effect.

Estimating coefficients in structural mean models can be done by defining a set of unbiased estimating equations. For the simple SMM (9) the solution is equal to the Wald estimator.^47^ This amounts more generally to G‐estimation.^23^, ^66^

Many authors apply 2SLS methods to binary outcomes by fitting linear regression outcome models and hence yielding estimates of risk differences. This is not advisable when also including covariates ***L*** as the fitted model may predict outcome values >1 or <0. Extending the two‐stage approach to a logistic regression outcome model is hampered by the nonlinearity of the logistic model. A two‐stage approach with a linear model in the first stage and a logistic model in the second stage can only be used to obtain IV estimates of odds ratios if the outcome is rare. Otherwise, an alternative may be to use logistic structural mean models.^59^, ^67^, ^68^

#### 4.2.4 When are IV methods useful?

We have discussed the IV assumptions needed to estimate causal treatment effects. Although many IV estimators are consistent, in finite samples instrumental variable estimators are generally biased. The bias depends on the sample size and on the strength of the instrument (ie, the correlation between *Z* and *A*).^69^ Furthermore, IV estimates are very sensitive to deviations from the IV assumptions. A small association between the unmeasured confounders and the instrument can lead to substantial bias especially if the instrument is weak.^57^, ^69^ Moreover, weak instruments yield very imprecise IV estimates and often (very) large sample sizes are needed to obtain informative results.^70^ This implies that instruments should be strongly correlated to the treatment. There is however a trade‐off between the amount of unmeasured confounding and the strength of the instrument: an instrument cannot be strong if there is substantial unmeasured confounding^57^ and a strong instrument implies weak unmeasured confounding.

To summarize, an instrumental variable analysis may be useful in the following situations: (1) the amount of expected unmeasured confounding is substantial, (2) an instrument exists for which the core IV assumptions are plausible and additionally a fourth assumption to interpret the point estimate can be sensibly invoked, (3) the instrument is sufficiently strong, and (4) sample sizes are sufficiently large (when instruments are weak, required sizes may be in the order of several thousands of subjects). Otherwise methods assuming NUC should be considered, while also maximizing the number of measured confounders. Although approaches relying on NUC yield biased estimators if unmeasured confounding is present, the direction of the bias is often known and the size of the bias may be approximated in sensitivity analyses.

### 4.3 Choosing an estimation method

Table 3 reviews several points that go to the heart of which causal estimands are meaningful and relevant in the specific setting represented by our case study. An accompanying Table 4 summarizes the main assumptions that are invoked by the various methods reviewed in this section when aiming to estimate the ATE (in addition to no interference and causal consistency). The table is self‐explanatory and highlights that the core difference lies in whether we are prepared or not to assume NUC, given a vector of measured confounders ***L***. However it is worth stressing these additional points.

**TABLE 3:** Considerations for the ATE for exposures *A*_1_, … , *A*_4_; the same issues arise in estimation of the ATT and ATNT

Exposure	Estimand	Comments
*A*_1_	ITT effect	Randomization ensures unbiased estimation using simple contrasts
*A*_3_	ATE|*A*_1_ = 1, or ATE|*A*_1_ = 0	Effect of starting breastfeeding in a world where all (or no) women are offered the program. If we do not condition on *A*_1_, then we mix the two populations (or two “worlds”), which would never coexist outside of a trial where only half of women are offered the intervention. Furthermore, *A*_2_ is an effect modifier. Thus, correct specification of the outcome model requires an *A*_2_*A*_3_ term, and the ATE must then marginalize over the distribution of *A*_2_. Note that the conditioning on *A*_1_ is not relevant for estimating the causal effect of *A*_2_, as *A*_1_ has the role of an instrument for *A*_2_, but not for *A*_3_ or indeed for *A*_4_
*A*_4_	ATE|*A*_3_ = 1	There is no support in the data for an effect of *A*_4_ in women with *A*_3_ = 0. Note also that *A*_4_ = 0 is a mixture of durations of breastfeeding, potentially from 1 day up to just shy of 3 months. The consistency assumption implies that its estimated effect refers to settings with the same distribution of breastfeeding discontinuation times. An equivalent statement holds for the interpretation of *A*_3_ = 0 in the row above

Abbreviation: ITT = Intention‐to‐treat.

**TABLE 4:** Sufficient assumptions for estimation methods of the ATE of a binary single point exposure *A* (assuming throughout that no interference and consistency hold)

	Assumptions				
		Correct specification of			
Method	NUC	*Y* model	PS^a^ model	Core IV assumption	No *Z*‐*A* interaction
Outcome regression			
conditional on *L*	✓	✓		
conditional on *PS* = *e*(***L***)	✓	${✓}^{a}$	${✓}^{a}$	
Stratification by *e*(***L***)	✓		✓	
Matching by *e*(***L***)	✓		✓	
IPW by *e*(***L***)	✓		✓	
DR via ***L*** and *e*(***L***)	✓	Either	Or		
IV *Z*				✓	✓

^a^Either of these if the outcome model is linear.

For those methods assuming NUC:

- Outcome regression assumes a correct specification of the outcome model.
- PS‐matching and PS stratification assume that the PS balances the confounder distribution.
- IPW assumes that the PS model is correctly specified given a sufficient set of confounders.
- Linear outcome regression models that condition on the estimated PS, as opposed to the original vector of confounders ***L***, require that either the outcome model or the PS model is correct and that the treatment effect does not vary with the PS.^71^
- The specification of the PS model should achieve balancing of the distribution of the measured confounders across treatment arms. Achieving this aim is substantially different from achieving treatment prediction, and hence the criteria used for the latter do not apply here.
- In general, outcome regression is more efficient than a PS‐based method.
- The choice between PS‐based methods (ie, stratifying, regression adjustment, matching, and IPW) depends on efficiency is an issue. Weighting may be inefficient (unless a doubly robust approach is used) if there are subjects with a very high or low PS value; matching has a trade‐off between a close match (which implies loss of efficiency because not all subjects are matched) vs residual confounding. PS‐regression adjustment has the advantage that it is robust against misspecification of the outcome model when the PS model is correctly specified. It can also be made more efficient with the inclusion of a selection of elements in *L*.^23^

When not assuming NUC

- IV estimation replaces the NUC assumption with other rather stringent assumptions.
- IV methods yield estimates that are very inefficient when instruments are weak and suffer from small sample bias.^69^

With any given approach come choices in implementation that imply a trade‐off between bias and variance. For example, in the context of PS matching, the use of smaller calipers to determine a match will reduce bias but may lead to a smaller matched sample and hence loss in efficiency. In PS‐inverse weighting, the use of weight truncation to reduce the influence of a small number of points has the effect of decreasing the variance at the cost of introducing some bias. It is hence impossible to recommend a single “best” approach, but rather choices are specific to the context where researchers must balance bias, statistical efficiency, and in some cases computational efficiency.

## 5 RESULTS FROM THE SIMULATION LEARNER

We applied the methods discussed in the previous section to estimate the ATE and the ATT of *A*_1_, *A*_2_, and *A*_3_ on weight at 3 months using the data from the simulation learner PROBITsim. More details and the code used to produce the reported results are given in Appendix 2 and in the material available at www.ofcaus.org.

### 5.1 Effect of the randomized program offer (*A*_1_)

First we estimate the causal effect of the randomized offer of the BEP (*A*_1_) on weight at 3 months. This is simply the difference in mean weight at 3 months between those with *A*_1_ = 1 and *A*_1_ = 0 because *A*_1_ is randomized. This is also an estimate of the intention‐to‐treat (ITT) effect, in this case an “intention to educate,” and is most relevant for health policy makers. This estimate is 94.2 g (95% confidence interval: 76.4 to 112.0 g). It indicates that inviting all expecting mothers in the study population to attend this specific program increases their baby's weight, on average, by 94 g. The true value obtained from Table 2 was 98 g and is well within the confidence interval.

### 5.2 Effect of program uptake (*A*_2_)

Table 5 shows the estimated ATE for *A*_2_, which is the effect most directly relevant to women deciding whether or not to attend the program if offered. We also show the corresponding estimated ATT. In Section 3 we showed that the true ATE_2_ was greater than ATT_2_ (165.1 vs 152.8 g), whereby the treated, that is, the mothers who attended the program, were on average, more educated and their infants had higher weight at 3 months but smaller increases from attending the program. We estimated these target parameters under different assumptions and model specifications, starting from crude estimates where confounding is ignored (${\hat{\text{ATE}}}_{2}=196.0$ g and ${\hat{\text{ATT}}}_{2}=148.7$ g). We then controlled for measured confounding via outcome regression, adopting two alternative model specifications that included all the potential confounders for the *A*_2_ to weight at 3 months relationship: maternal age, education, allergy status, smoking during pregnancy, and area of residence. In the first specification we included a quadratic term for maternal age, and in the second we also included interactions between *A*_2_ and each confounder. The first led to ${\hat{\text{ATE}}}_{2}=155.4$ g and the second to ${\hat{\text{ATE}}}_{2}=165.0$ g, much closer to the true value of 165.1 g.

**TABLE 5:** Estimated ATE and ATT of *A*_2_ on weight at 3 months (in grams) obtained using alternative estimation methods controlling for relevant confounders^a^; PROBITsim study

Estimand	Estimation method	Estimate (SE)
ATE
	True value	165.1
	Crude regression	196.0 (9.6)
	Regression adjustment (without interactions)	155.4 (9.5)
	Regression adjustment (with interactions)	165.0 (9.7)
	PS stratification^b^ (six strata)	165.0 (9.4)
	Regression with PS^b^	156.2 (9.0)
	PS matching (one match)^c^	155.7 (10.1)
	PS matching (three matches)^c^	154.9 (10.1)
	PS IPW^b^	164.7 (9.3)
	PS DR IPW^b^	164.7 (9.7)
	IV	146.2 (14.0)
ATT
	True value	152.8
	Regression adjustment (with interactions)	148.7 (9.4)
	PS stratification^b^ (six strata)	148.7 (9.6)
	PS matching (one match)^c^	145.8 (9.8)
	PS matching (three matches)^c^	145.4 (9.7)
	PS IPW^b^	148.0 (9.6)

^a^The variables controlled for in each of these analyses were: maternal age (linear and quadratic term), maternal education, maternal allergy status, smoking status in the first trimester (ie, before program allocation), and area of residence.

^b^SE estimated by bootstrap with 1000 replications.

^c^SE estimated according to Abadie and Imbens (2012), assuming that the conditional outcome variance is homoscedastic, that is, does not vary with the covariates or treatment. This is implemented in Stata with the option vce(iid). This assumption can be relaxed using the option vce(robust, nn(2)) for the one match analysis and vce(robust, nn(4)) for the three matches analysis.

When applying the PS‐based methods, we fitted the PS model by logistic regression with the same confounders (including the quadratic term for maternal age). Stratification (over six strata) led to the same estimates as the more general outcome regression models (${\hat{\text{ATE}}}_{2}$ = 165.0 and ${\hat{\text{ATT}}}_{2}=148.7$ g), while matching, either to 1 or 3 other infants, led to slightly smaller and less precise estimates. Balance checks revealed that the PS model was well specified (see Appendix 2). Adopting inverse weighting or doubly robust estimation gave point estimates and standard errors close to those from outcome regression.

The reported IV estimate used *A*_1_ as the instrument and assumed no *A*_1_–*A*_2_ interaction to be interpreted as an ATE. This was estimated at 146.2 g and, as expected, has a very large estimated standard error.

### 5.3 Effect of starting breastfeeding (*A*_3_)

The estimated ATE and ATT for the effect of *A*_3_ on infant weight at 3 months are found in Table 6. As before they are obtained under different assumptions and using different methods. As their true values depend on whether the exposure is set in a world where the BEP is or not present, results are reported separately under these two scenarios.

**TABLE 6:** Estimated ATE and ATT of *A*_3_ on weight at 3 months (in grams) obtained using alternative estimation methods controlling for relevant confounders^a^ and stratified by whether mothers were offered the BEP program; PROBITsim study

		*A*_1_ = 0	*A*_1_ = 1
Estimand	Estimation method	Estimate (SE)	Estimate (SE)
ATE			
	True value	386.8	422.3
	Crude regression	503.2 (11.6)	582.0 (12.2)
	Regression adjustment (without interactions)	384.3 (2.8)	428.0 (3.3)
	Regression adjustment (with interactions)	384.7 (3.3)	425.3 (2.7)
	Regression with PS^b^	384.4 (3.2)	425.9 (3.3)
	PS stratification^b^ (6 strata)	392.2 (4.1)	442.0 (6.7)
	PS matching (one match)^c^	386.5 (13.7)	429.0 (17.4)
	PS matching (three matches)^c^	380.7 (12.4)	437.2 (15.2)
	PS IPW^b^	384.7 (4.0)	426.6 (6.9)
	PS DR IPW^b^	384.8 (3.9)	426.7 (7.1)
ATT			
	True value	380.1	421.4
	Regression adjustment (with interactions)	378.0 (2.9)	421.7 (2.5)
	PS stratification^b^ (six strata)	388.8 (5.1)	438.3 (9.5)
	PS matching (one match)^c^	384.3 (15.8)	435.6 (21.2)
	PS matching (three matches)^c^	387.9 (13.5)	441.2 (18.0)
	PS IPW^b^	381.9 (5.1)	429.2 (10.1)

^a^The variables controlled for in each of these analyses were: maternal age (linear and quadratic term), maternal education, maternal allergy status, smoking status in the first trimester (ie, before program allocation), area of residence, baby's birth weight (linear and quadratic term), whether birth was by caesarian section and, in the analyses restricted to *A*_1_ = 1, whether the mother attended the program.

^b^SE estimated by bootstrap with 1000 replications.

^c^SE estimated according to Abadie and Imbens (2012), assuming that the conditional outcome variance is homoscedastic, that is, does not vary with the covariates or treatment. This is implemented in Stata with the option vce(iid). This assumption can be relaxed using the option vce(robust, nn(2)) for the one match analysis and vce(robust, nn(4)) for the three matches analysis.

Note also that the true average potential outcome in the world where no program was offered but all mothers start breastfeeding was lower than in the world where BEP is offered to all mothers and they all start breastfeeding (Table 6, rows 8 and 9) because of the effect of the BEP on breastfeeding duration. This impacts on the causal effect of breastfeeding: when *A*_1_ is set at 0, that is, no BEP is available to anyone, the effect of starting breastfeeding is ${\text{ATE}}_{3,a_{1}(0)}=386.8$ g and ${\text{ATT}}_{3,a_{1}(0)}=380.1$ g; while when *A*_1_ set to 1, ${\text{ATE}}_{3,a_{1}(1)}=422.3$ g and ${\text{ATT}}_{3,a_{1}(1)}=421.4$ g.

The confounders of the *A*_3_ to weight at 3 months relationship include not only maternal age, education, allergy status, smoking during pregnancy, and area of residence (ie, those involved in the analyses of *A*_2_) but also the infant's sex, birth weight (including a quadratic term), and whether the infant was born by caesarian section. In the analyses concerning the world where *A*_1_ is set to be 1, *A*_2_ is also a confounder as it influences both *A*_3_ and infant weight.

There is little difference across the ATE estimates, obtained using either outcome regression or PS‐based methods: the results are all very similar and standard errors, while variable, all still lead to the conclusion that *A*_3_ meaningfully and statistically affects the outcome. Balance checks for these two scenarios revealed that the PS model was relatively well specified in both, and there was good overlap in propensity of exposure between the groups defined by *A*_2_ and *A*_3_ (see Appendix 2).

We do not produce an equivalent IV estimate as there is no suitable IV for this effect, since *A*_1_ violates the second IV assumption: *A*_1_ influences the outcome not only via *A*_3_ but also via *A*_2_.

For the ATT estimates, regression adjustment seems to perform better than the other methods, especially in the world where *A*_1_ = 1. Of course, our simulation learner has generated just one relatively simple world model where both our outcome and propensity model are easy to specify.

## 6 DISCUSSION

We set out to discuss “the making of” a causal effect question involving a well‐defined point exposure for which we seek to find the average treatment effect, possibly conditional on baseline characteristics. We have maintained an emphasis on the framing of the scientific causal question, and in considering many methods together, in their basic form, so as to compare and contrast the required assumptions of different principled estimation approaches for directly targeted estimands.

We applied the concept of principled estimation in turn to four different options for exposure levels which present themselves along the path from treatment prescription to completion. As we moved with the selected exposure along this path, the sufficient set of baseline confounders (and effect modifiers) became richer, and we had to account for what happened earlier in the path. In doing so, we saw that we cannot treat randomization as “once an instrument, always an instrument.” Rather, randomization (our *A*_1_) may act as an instrument for the effect of following the program (*A*_2_), but it violated the assumptions required for it to be an instrument when studying the effect of “starting breastfeeding” (*A*_3_). At every instance, thought is required to adapt to the new situation and estimate a relevant causal effect in a (sub)population of interest.

In a similar vein, confounders that act as effect modifiers could be conditioned on to estimate average causal effects within specific population strata (or by including interaction terms). Subsequently, we can average over their distribution in the population of interest. With additional averaging, we lose some ability to offer stratified evidence and provide personalized information but uphold a more global public health perspective. This pertains to both the ATE and ATT target.

For selected estimands, we showed how the various estimating approaches perform in their most basic form. We recognized that many of them operate under similar identifying assumptions. For example, the different propensity methods all assume correct specification of the PS model, and when choosing one of the methods one should consider additional issues. For the stratification, the choice of the number of strata and residual bias, for the matching the trade‐off between finding matched individuals and the fineness of the matching, and for the inverse probability weighting, the size of the weights, truncation. Of course, differences remain in operating characteristics when key (untestable) assumptions are violated. The list of available approaches under the NUC assumption includes familiar standardized means derived from the classic regression of outcome on baseline covariates and the exposure. This need not perform worse, and can even be better than more novel PS‐based methods that seek covariate balance after using the propensity score for regression, matching, stratification, or inverse probability weighting. Doubly robust methods may be expected to outperform others when one set of model assumptions is violated, but equally loses precision (increases error) when both the outcome regression and PS model are ill‐fitting, and may be inefficient in finite samples when only one model is correctly specified.^65^

When we cannot find a sufficient set of confounders, instrumental variable approaches form an appealing alternative provided an instrument can be found. To interpret the resulting estimator additional assumptions are needed that are not always easy to justify; and one should consider whether those can lead to very broad confidence intervals. There are other alternative routes still, such as regression discontinuity approaches for instance,^72^ a variation on pseudorandomization that is found in specific designs.

We set out to give an overview of the basic principles that guide causal inference, however in practice, many complications conspire to challenge the applied statistician when performing causal inference. We, for instance, have implicitly assumed all covariates are measured without error and there is no selection bias or drop‐out. In practice, data may be not just confounded, but may also suffer from missingness^31^ and measurement error on exposure^73^ or confounders^74^ is likely. Flexible models may be more appropriate to capture the associations involved. Clustering and no‐interference may require extension of the presented setup to incorporate interference.^12^, ^13^, ^75^ With substantial dropout from a longitudinal outcome due to mortality, one must adapt the definition of the outcome explicitly or reduce the target population to potential survivors on all treatments considered.^76^ In the international initiative of Strengthening Analytical Thinking for Observational Studies (STRATOS),^77^ other topic groups focus on guidance for these topics and joint developments with our causal inference topic group are envisaged for the future.

We have purposefully focused on the point (ie, fixed) exposure perspective, even though we considered a natural sequence of such exposures with corresponding decisions to be made. This allowed us to present an overview of different estimation principles, showing how they resemble one another, and where and how they differ in their fundamental assumptions and performance. The natural next step is to consider the joint effect of a sequence of exposure options $a_{2},a_{3},a_{4}$ as a time‐varying treatment regime and engage in estimating causal effects of different (static or dynamic) treatment strategies. To achieve this, we would need to formally account for time‐varying confounders along that path (see, eg, References 78, 79). We might further aim to explain the total effect and engage in mediation analysis to evaluate the possible role of intermediate variables on the causal path.^71^, ^80^, ^81^ For all these endeavors in higher dimensions, the principles laid out here continue to form an important foundation.

Even at the point exposure level, the literature on adaptations of these estimators under additional or alternative assumptions is vast, but beyond the scope of this tutorial. Here, we focused on a binary exposure and a continuous, uncensored outcome. When exposures are categorical or continuous, a *generalized* propensity score can be used.^82^, ^83^

There is course material available that accompanies this article, where practical exercises discuss estimation when the primary outcome is binary, using the Right Heart Catheterisation data set^84^ (www.ofcaus.org). Estimating a linear effect, a risk difference, is less obvious there and requires extra care.

We hope the layout of this principled approach will inspire practicing statisticians to think carefully about what they are estimating and to report as clearly as possible on the nature of their exposure and causal estimand, as well as the assumptions on which they have relied. While an abundance of machine learning techniques can handle electronic health records, they too need to integrate fundamental principles of causal inference to address causal questions.^85^ A naive analysis can be dangerous when followed by either implicit or explicit causal claims that are made without regard for confounding or effect modification or for their population‐level interpretation. We hope this contribution can generate confidence and insight into methodological ground‐rules, and promote better thinking, reliable estimates, and clear reporting.

## Supporting information


**Data S1**. Appendix 1

**Data S2**. Appendix 2

## Data Availability

The simulation and analysis code that supports the findings of this study are available at the following publicly accessible websites: www.ofcaus.org and the linked GitHub depository https://github.com/IngWae/Formulating‐causal‐questions

## Footnotes

1 For those familiar with the longitudinal, time‐varying exposure outcome setting, this is a *marginal structural model*.^3^ Of course note that the ATE itself targets a marginal structural mean contrast, either through direct modeling of the mean as accomplished via inverse weighting, or by modeling a conditional structural model and then marginalizing over the covariate distribution as in regression outcome modeling.
