# Supplementary material for: Formulating causal questions and principled statistical answers
Source: Stat Med. 2020 Sep 23;39(30):4922–48. doi: 10.1002/sim.8741 (PMC7756489; doi:10.1002/sim.8741)
Supplement: Supplementary file 1 — Data S1. Appendix 1 [file SIM-39-4922-s001.pdf]

Appendix 1 — Generation of the *simulation learner* PROBITsim

1 Introduction

In the companion paper we discuss the estimation and interpretation of various estimands using simulated data. The generation of these data was informed by a real investigation but enriched here by the generation of potential outcome data, in addition to factual data. We follow Wallace et al (2015) in simulating data inspired by the results of the Promotion of Breastfeeding Intervention Trial (PROBIT) (Kramer et al, 2001). In this trial mother-infant pairs across 31 Belarusian maternity hospitals were cluster randomised to receive either standard care or a breastfeeding encouragement intervention to investigate the effect of breastfeeding on a child's later development. In our simulation we are randomising individual mother-infant pairs and are focusing on weight achieved at age 3 months, thus the study population is babies that survive the first three months.

The DAG in Figure 1 sketches the underlying causal relationships between the simulated variables.

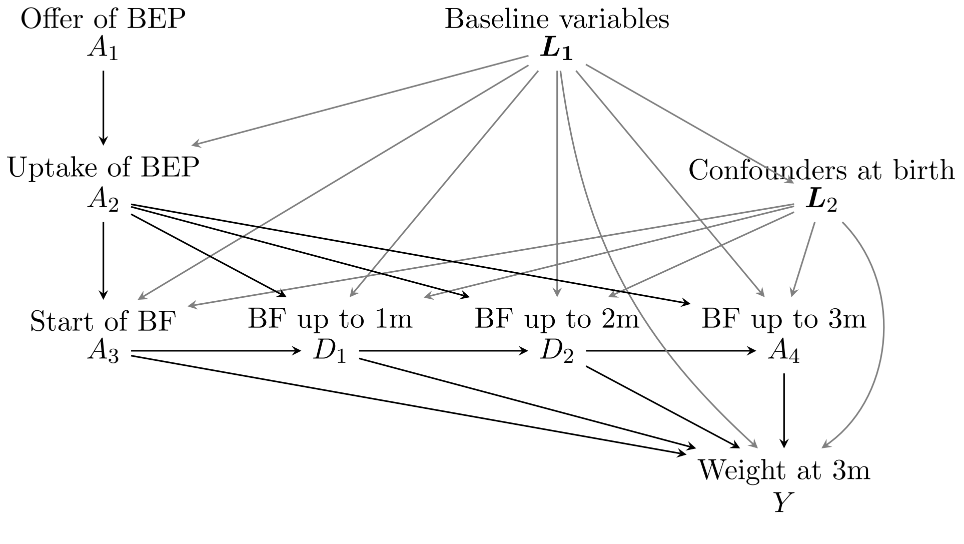

Figure 1: Causal diagram of the data generating model for the Simulation Learner. BEP: breastfeeding encouragement programme; BF: breastfeeding; m: months

In the next sections we describe the models we used to simulate the data, called PROBITsim.

2 The baseline variables

The distribution of baseline variables, $\boldsymbol{L}$, was made to resemble that of the Belarus study. In all simulations, binary variables were generated from the binomial distribution, and categorical variables with more than two categories were generated using a multinomial distribution.

- The sample size n is set to be 17,044.
- Women can live at four different locations (1: urban western region, 2: rural western region, 3: urban, eastern region and 4: rural, eastern region) with frequency distribution (0.33,0.16,0.26,0.25).
- Age is assumed to be log normal $\log(age) \sim N(3.17, 0.19)$. If the simulated age ≤ 13 years, it is set to be 13 years. This yields a median age of 24 years (interquartile range 21-27 years).
- The child's sex (Sex) is a binary variable. Boys are coded as 1, girls as 0. The probability to be male is 52%.
- Education (Educ) had 3 levels (1=low, 2=medium, 3=high). Its distribution depends on the location, according to the Belarus study, where the probability of having low, medium or high education is set as:
  - at location 1: low: 0.31, medium 0.54, high 0.15
  - at location 2: low: 0.44, medium 0.44, high 0.12
  - at location 3: low: 0.33, medium 0.51, high 0.16
  - at location 4: low: 0.41, medium 0.48, high 0.11
- There are several baseline variables that depend on education level:
  - Smoking during pregnancy (Smoke) is a binary variable, with probability of a positive value set to be equal to 0.40 for low education, 0.25 for medium education and 0.10 for more highly educated women.
  - Maternal allergy (Allergy) is a binary variable. The probability of having a mother member who suffers from allergy is 0.03 for low education, 0.05 for medium education and 0.07 for more highly educated women.
  - Born by caesarian section (Caesarean) is a binary variable. The probability of a caesarean birth is set to be equal to 0.10 for mothers with low education, 0.12 for medium education and 0.16 for more highly educated women.
- Birth weight (Wgt0) is normally distributed and its mean (E) depends on the child's sex, maternal smoking and education. The standard deviation (SD) is set to be larger for boys than for girls.

  E(Wgt0) =2950 + 140 Sex + 80 (Educ=2) + 160 (Educ=3) - 200 Smoke
  SD(Wgt0) = 390 + 30 Sex

  where we use the shorthand $(X = x)$ for $I_{(X=x)}$, the indicator that the statement within parentheses is true.

Table 2 provides a summary of these data.

3 Potential and observed exposures

We consider a randomised trial where randomly half of the pregnant women received an intervention that consisted of an offer for a breastfeeding encouragement programme. We assume than only women in the intervention group have access to the encouragement programme. In this study, we distinguish four different exposure types of interest:

- $A_1 = 1$: being assigned to the intervention group, in which the encouragement programme is offered; $A_1 = 0$: otherwise.

- $A_2 = 1$: actually taking up the training offer (e.g. a course followed, literature read); $A_2 = 0$: otherwise.

- $A_3 = 1$: starting breastfeeding; , $A_3 = 0$: otherwise.

- $A_4 = 1$: starting breastfeeding and continuing for the full 3 months;, $A_4 = 0$: otherwise.

For each woman in the study, we generated potential exposure values for A_2, A_3 and A_4 when setting A_1 (and in some instances A_2) to be 1 or to 0. The following potential exposures were generated: .

- $A_{2,\mathfrak{a}_1(1)}$ and $A_{2,\mathfrak{a}_1(0)}$, where $A_{2,\mathfrak{a}_1(1)}$ represents the potential exposure A_2 when A_1 is set to take the value 1, and similarly for $A_{2,\mathfrak{a}_1(0)}$. These potential exposures indicate whether the training programme would be taken up, had A_1 been set to be 1 or 0.

- $A_{3,\mathfrak{a}_1(1)}$ and $A_{3,\mathfrak{a}_1(0)}$. They indicate whether breastfeeding would be initiated if the breastfeeding programme had been offered (A_1 set to 1 being denoted $\mathfrak{a}_1(1)$) or not ($\mathfrak{a}_1(0)$).

- $A_{3,\mathfrak{a}_2(1)}$, $A_{3,\mathfrak{a}_2(0)}$. They indicate whether breastfeeding would be initiated and continued for 3 months, had A_2, the training, been set to be 0 or 1.

Because we assumed that the programme is only available to women in the intervention group, women in the control group have no access to it, i.e. $A_{2,\mathfrak{a}_1(0)} = 0$ for all women. This also implies that $A_{3,\mathfrak{a}_1(0)} = A_{3,\mathfrak{a}_2(0)}$.

The next sections describe how these potential exposure realisations and the observed data were generated.

3.1 A_1: randomised intervention

In our simulation women are randomly assigned to receive the offer of the breastfeeding encouragement programme (BEP), or standard care.

- The intervention (A_1) is a binary variable with $Pr(A_1 = 1) = 0.50$.

3.2 A_2: the programme offer is actually taken up

When the programme is offered, a subgroup of women will take up the invitation and will actually follow the programme. We assume that the more highly educated women are more inclined to follow the programme. For each woman we generated the potential variable $A_{2,\mathfrak{a}_1(1)}$ indicating whether the woman would have followed the programme had she been randomised to the intervention arm. We use a logistic regression model to relate the odds of following the programme to maternal age, education and smoking status during pregnancy as follows:

$$Pr(A_{2,\mathfrak{a}_1(1)} = 1) = \text{expit(-1.9+ 0.1 Age + 0.5 (Educ = 2)+1.0 (Educ = 3)-1.0 Smoke)}.$$

The potential variable $A_{2,\mathfrak{a}_1(0)}$ is 0 for all women because it is only possible to follow the programme after receiving an invitation for it (i.e. when A_1=1).

Assuming that the consistency assumption holds, the observed treatment A_2 is then

$$A_2 = \begin{cases} A_{2,\mathfrak{a}_1(1)} & \text{if } A_1 = 1 \\ 0 & \text{if } A_1 = 0 \end{cases}$$

3.3 A_3: the mother actually starts breastfeeding

We generated an ordinal variable X, representing an unmeasurable individual characteristic, to distinguish three different types of women: women who would always start breastfeeding whether the BEP is offered or not ($X = 2$), women who would start breastfeeding after following the encouragement programme, but would not, if the programme were not followed (X=1), and women who would never start breastfeeding ($X = 0$). This variable is used to generate the potential breastfeeding behaviour under different values of the intervention and potential programme uptake but will be treated as an unobservable individual characteristic (and referred to as "principal strata"). We assume that there are no women who will not breastfeed after following the programme, but will start breastfeeding, without following the programme, i.e. there are no defiers and the assumption of monotonicity holds.

The ordinal variable X was generated using an ordinal logistic model with:

- $Pr(X = 2)$ = expit (-2.5 + 0.25 (Educ =2) + 0.5 (Educ =3) + 0.1 Age + 0.008 Sex -0.5 Smoke + 0.0006 Wgt0).
- $Pr(X \geq 1)$ = expit(1.5 + logit (Pr(X=2))

This ordinal variable X was used to obtain the third treatment variable A_3. Three potential treatment outcomes were generated: $A_{3,\mathfrak{a}_1(1)}$ indicating whether a woman would start breastfeeding if randomised to the intervention arm, $A_{3,\mathfrak{a}_1(0)}$ whether she would start breastfeeding if randomised to the control arm, and $A_{3,\mathfrak{a}_2(1)}$, if she would start breastfeeding after following the programme. We assume that only women with $A_{2,\mathfrak{a}_1(1)} = 1$, i.e the women who would follow the programme if offered, could also be *compliers* to starting breastfeeding.

- $A_{3,\mathfrak{a}_1(1)} = 1$ if $X = 2$ or $X = 1$ and $A_{2,\mathfrak{a}_1(1)} = 1$, 0 otherwise
- $A_{3,\mathfrak{a}_1(0)} = 1$ if $X = 2$, 0 otherwise
- $A_{3,\mathfrak{a}_2(1)} = 1$ if $X = 2$ or $X = 1$, 0 otherwise
- $A_{3,\mathfrak{a}_2(0)} = 1$ if $X = 2$, 0 otherwise

Because we assumed that women in the control group had no access to the encouragement programme, $A_{3,\mathfrak{a}_1(0)} = A_{3,\mathfrak{a}_2(0)}$ for all women.

The observed treatment A_3 is then

$$A_3 = \begin{cases} A_{3,\mathfrak{a}_1(1)} & \text{if } A_1 = 1 \\ A_{3,\mathfrak{a}_1(0)} & \text{if } A_1 = 0 \end{cases}$$

Table 1 shows some of the data for the first 10 women. For example woman 1 has the intermediate level of education ('medium'). She is randomised to intervention ($A_1 = 1$), but because $A_{2,\mathfrak{a}_1(1)}$ is equal to 0 she does not actually follow the programme. The unmeasurable individual characteristic X was 0, indicating that the woman would not start breastfeeding either when randomised to the intervention or when randomised to control and so both $A_{3,\mathfrak{a}_1(1)}$ and $A_{3,\mathfrak{a}_1(0)}$ are 0. If counter to the facts she would have followed the programme, then she still would not have started breastfeeding ($A_{3,\mathfrak{a}_2(1)} = 0$) because $X = 0$. In practice we do not observe all these potential outcomes, but for this woman we observe $A_2 = 0$ and $A_3 = 0$.

Figure 2 illustrates the data generating mechanism for A_2 and A_3.

3.4 Description of the generated confounders and early exposure data

The following properties hold in our simulated population.

- The probability of following the programme when offered, $\Pr(A_{2,\mathfrak{a}_1(1)} = 1) = 0.64$.

Table 1: Generated potential exposure values of the first 10 women, PROBITsim Study.

	Educ	A_1	$A_{2,\mathfrak{a}_1(1)}$	X	$A_{3,\mathfrak{a}_1(1)}$	$A_{3,\mathfrak{a}_1(0)}$	$A_{3,\mathfrak{a}_2(1)}$	A_2	A_3
1	2	1	0	0	0	0	0	0	0
2	2	1	0	0	0	0	1	0	0
3	1	1	0	2	1	1	1	0	1
4	3	1	0	0	0	0	1	0	0
5	2	1	0	0	0	0	0	0	0
6	2	0	0	0	0	0	0	0	0
7	3	0	1	1	1	0	1	0	0
8	1	1	1	1	1	0	1	1	1
9	2	1	1	0	0	0	0	1	0
10	2	0	1	2	1	1	1	0	1

Educ is education: 1=low, 2=medium, 3=high; X is the unmeasurable individual characteristic, which distinguishes the different principal strata (never breastfeeding (X=0), always breastfeeding (X=2), and only starting breastfeeding if (A_2 =1 and $A_1 = 1$; X=1).

- The frequency distribution of the principal breastfeeding strata is 0.32 for never starters, 0.19 for compliers (they will start breastfeeding if randomised for the programme and not if they are in the control arm), 0.49 always starters.
- The probability of starting breastfeeding if randomised to control is $Pr(A_{3,\mathfrak{a}_1(0)} = 1) =$ 0.49.
- The probability of starting breastfeeding if randomised to intervention is $Pr(A_{3,\mathfrak{a}_1(1)} = 1)$ = 0.68.
- The probability of starting breastfeeding if the programme were followed by everyone $Pr(A_{3,\mathfrak{a}_2(1)} = 1) = 0.79$.

Table 2: Mean of baseline variables in the different treatment groups, PROBITsim Study, $N = 17,044$

		Intervention group						Control group			
Variable	Overall	$A_1 = 1$	$A_1 = 1$ $A_2 = 1$	$A_1 = 1$ $A_2 = 0$	$A_1 = 1$ $A_3 = 1$	$A_1 = 1$ $A_3 = 0$	$A_1 = 1$ $A_4 = 1$	$A_1 = 0$	$A_1 = 0$ $A_3 = 1$	$A_1 = 0$ $A_3 = 0$	$A_1 = 0$ $A_4 = 1$
Location= 1	0.33	0.32	0.33	0.31	0.33	0.31	0.33	0.34	0.34	0.34	0.35
Location= 2	0.16	0.17	0.16	0.17	0.17	0.17	0.16	0.16	0.17	0.15	0.17
Location= 3	0.26	0.26	0.26	0.26	0.27	0.25	0.27	0.27	0.26	0.27	0.26
Location= 4	0.24	0.25	0.24	0.26	0.24	0.28	0.24	0.23	0.23	0.24	0.22
Age	24.3	24.3	24.9	23.1	25.0	22.7	25.3	24.3	25.3	23.2	25.6
Educ= low	0.36	0.36	0.3	0.48	0.32	0.45	0.25	0.35	0.31	0.39	0.21
Educ= medium	0.50	0.50	0.53	0.45	0.52	0.46	0.56	0.51	0.52	0.50	0.57
Educ= high	0.14	0.14	0.17	0.07	0.16	0.09	0.19	0.14	0.17	0.11	0.21
Smoke	0.27	0.27	0.19	0.43	0.21	0.40	0.18	0.27	0.20	0.34	0.18
Allergy	0.04	0.04	0.05	0.04	0.04	0.04	0.05	0.05	0.05	0.04	0.06
Caesarean	0.12	0.12	0.12	0.11	0.12	0.11	0.10	0.12	0.13	0.12	0.09
Birth weight	3028	3024	3049	2979	3070	2927	3111	3032	3099	2967	3158
Sex	0.52	0.52	0.52	0.52	0.54	0.48	0.52	0.51	0.52	0.51	0.50

3.5 A_4: the mother starts breastfeeding and continues for the full 3 months

To define this fourth variable we have to model the duration of breastfeeding. We do so in the next section.

4 Potential duration of breastfeeding in the first three months

The duration of breastfeeding varies between mothers and depends on education, birth weight, allergy, age of mother, sex of child, caesarean section, and on whether the woman did follow the BEP.

For each subject we started by generating the following two potential breastfeeding durations:

- $D_{\mathfrak{a}_2(0),\mathfrak{a}_3(1)}$, the potential breastfeeding duration if a woman had been set to start breastfeeding and not to follow the programme.
- $D_{\mathfrak{a}_2(1),\mathfrak{a}_3(1)}$, the potential breastfeeding duration if a woman had been set to start breastfeeding and to follow the programme.

For simplicity, we generated discrete duration variables, with values 0, 1, 2 and 3 months. Therefore we assumed an underlying truncated Poisson model for potential duration:

$$D_{\mathfrak{a}_2(a),\mathfrak{a}_3(1)} \sim min(3, Poisson(\lambda(a)))$$

with $\lambda(a)$=1.5+0.001 (Wgt0-3000) + 1.0 (Educ=2) + 1.5 (Educ=3) + a_2 + 0.5 Allergy + 0.05 Age -1.0 Caesarian -0.5 Sex, with a the value to which A_2 has been set. In this way following the programme does not only increase the probability of starting breastfeeding, but also increases the duration of breastfeeding if started.

These potential variables can be used to define the potential duration of breastfeeding when assigned to the intervention group:

$$D_{\mathfrak{a}_1(1)} = \begin{cases} 0 & \text{if } A_{3,\mathfrak{a}_1(1)} = 0 \\ D_{\mathfrak{a}_2(1),\mathfrak{a}_3(1)} & \text{if } A_{3,\mathfrak{a}_1(1)} = 1 \text{ and } A_{2,\mathfrak{a}_1(1)} = 1 \\ D_{\mathfrak{a}_2(0),\mathfrak{a}_3(1)} & \text{if } A_{3,\mathfrak{a}_1(1)} = 1 \text{ and } A_{2,\mathfrak{a}_1(1)} = 0. \end{cases}$$

The potential duration of breastfeeding when assigned to the control group is:

$$D_{\mathfrak{a}_1(0)} = \begin{cases} 0 & \text{if } A_{3,\mathfrak{a}_1(0)} = 0 \\ D_{\mathfrak{a}_2(0),\mathfrak{a}_3(1)} & \text{if } A_{3,\mathfrak{a}_1(0)} = 1. \end{cases}$$

The potential duration when assigned to attending the programme (and hence also when assigned to the intervention group) is

$$D_{\mathfrak{a}_2(1)} = \begin{cases} 0 & \text{if } A_{3,\mathfrak{a}_2(1)} = 0 \\ D_{\mathfrak{a}_2(1),\mathfrak{a}_3(1)} & \text{if } A_{3,\mathfrak{a}_2(1)} = 1 \end{cases}$$

The frequency distribution of the different potential duration variables is given in Table 3.

The actual observed duration of BF and the observed value of A_4, continuing breastfeeding for the full three months are equal to

- $D = \begin{cases} D_{\mathfrak{a}_1(1)} & \text{if } A_1 = 1 \\ D_{\mathfrak{a}_1(0)} & \text{if } A_1 = 0 \end{cases}$
- $A_4 = 1$ if $D \geq 3$ months and 0 otherwise.

Figure 3 illustrates the data generating mechanism for D and A_4.

Table 3: True potential duration of breastfeeding under different scenarios, generated as in PROBITsim Study, but with $N = 5,000,000$

Duration	Scenario	Frequency distribution Months of Breast Feeding 0	1	2	≥ 3
$D_{\mathfrak{a}_1(0)}$	No intervention	0.54	0.07	0.09	0.30
$D_{\mathfrak{a}_1(1)}$	Intervention offered	0.34	0.06	0.10	0.50
$D_{\mathfrak{a}_2(1)}$	Programme offered and followed	0.22	0.06	0.11	0.61
$D_{\mathfrak{a}_1(0),\mathfrak{a}_3(1)}$	Programme not offered, BF started	0.07	0.15	0.20	0.58
$D_{\mathfrak{a}_2(1),\mathfrak{a}_3(1)}$	Programme followed, BF started	0.02	0.08	0.14	0.75

5 Potential outcomes for weight at three months

We generated the potential weight at 3 months ($Y(\mathfrak{D})$) under different potential durations of breastfeeding (D) using a linear regression model that included birth weight and all the other baseline variables, duration of breastfeeding during the first 3 months of life, and an interaction terms between duration of breastfeeding with birth weight, education and maternal smoking, assuming that children with lower birth weight, children of less educated mothers and children with smoking mothers benefited more from breastfeeding. The model for potential birth weight, (in grams), under different set durations of breastfeeding, was specified as follows:

$E[Y(\mathfrak{D})]$ = 5800 + (Wgt0-3000) + 16 (Location=2) - 20 (Location=3) - 15 (Location=4) + 10 (Educ=2) + 20 (Educ=3) - 50 Smoke - 25 Allergy - 10 Age - 40 Caesarian + 500 Sex + 100 D + 50 D (Educ=2) + 100 D (Educ=1) - 0.02 D (Wgt0-3000) + 50 D Smoke

where D is the duration of breastfeeding generated under different set values for A_1 and A_2 as described in Section 4. Individual realizations were generated assuming a normal distribution with SD=50g (assumed to have a biological variation of sd =40 g and a residual component of sd=10g).

Since duration of breastfeeding varies according to intervention, uptake of the programme, and uptake of breastfeeding (i.e.A_1, A_2 and A_3), the potential outcomes under different scenarios that influence duration were calculated. Several potential outcomes $Y_{\cdot}$ were generated to represent the potential weight at 3 months under different interventions:

- $Y_{\mathfrak{a}_3(0)}$, the potential outcome under a no breastfeeding uptake (D=0).
- $Y_{\mathfrak{a}_1(0)}$, the potential outcome under no BEP intervention (no programme offer).
- $Y_{\mathfrak{a}_1(1)}$ the potential outcome under BEP intervention.
- $Y_{\mathfrak{a}_2(1)}$ the potential outcome under programme uptake.
- $Y_{\mathfrak{a}_1(0),\mathfrak{a}_3(1)}$ the potential outcome under a joint intervention where the programme is not offered and breastfeeding is set to start.
- $Y_{\mathfrak{a}_1(1),\mathfrak{a}_3(1)}$ the potential outcome under a joint intervention where the programme is offered and breastfeeding is set to start.
- $Y_{\mathfrak{a}_2(1),\mathfrak{a}_3(1)}$ the potential outcome if the programme is actually followed and breastfeeding is started.
- $Y_{\mathfrak{a}_4(1)}$, the potential outcome if breastfeeding is set to last for three months.

The mean and standard deviation of the different potential outcomes at three months in our simulated population are given in Table 4.

Table 4: True potential weight at three months (mean and standard deviation) in the study population under different scenarios, generated as in PROBITsim Study, but with $N = 5,000,000$.

Outcome	Scenario	Mean	SD
$Y_{\mathfrak{a}_3(0)}$	No BF	5826	533
$Y_{\mathfrak{a}_1(0)}$	intervention is not offered	6014	592
$Y_{\mathfrak{a}_1(1)}$	intervention is offered	6112	591
$Y_{\mathfrak{a}_2(1)}$	programme is followed	6178	576
$Y_{\mathfrak{a}_1(0),\mathfrak{a}_3(1)}$	no intervention, BF is started	6213	550
$Y_{\mathfrak{a}_1(1),\mathfrak{a}_3(1)}$	intervention, BF is started	6248	540
$Y_{\mathfrak{a}_2(1),\mathfrak{a}_3(1)}$	programme followed, BF is started	6275	528
$Y_{\mathfrak{a}_4(1)}$	duration BF = 3 months	6348	497

SD: standard deviation

The observed birth weight is generated according to the observed combination of values for A_1, A_2, and D. Figure 4 illustrates the corresponding data generating mechanism.

6 Different causal effects of interest

To explore the effect of different forms of interventions in different sub-populations, we calculated for each potential outcome the weight gain at 3 months, compared to a no breastfeeding scenario. The mean weight gain is calculated in different sub-populations, and results are given in Table 5. The table can be used to calculate all kinds of contrast of interest.

Table 5: Potential weight differences, under different conditions, compared to a no breastfeeding hypothetical intervention, in different populations generated as in PROBITsim Study, but with $N = 5,000,000$.

Outcome	Scenario	totpop	prog	noprog	BF.interv	no BF	compliers
$Y_{\mathfrak{a}_3(0)}$	No BF	0	0	0	0	0	0
$Y_{\mathfrak{a}_1(0)}$	intervention is not offered	187	195	175	277	0	0
$Y_{\mathfrak{a}_1(1)}$	intervention is offered	286	348	175	422	163	437
$Y_{\mathfrak{a}_2(1)}$	programme is followed	351	348	355	437	274	437
$Y_{\mathfrak{a}_1(0),\mathfrak{a}_3(1)}$	no intervention, BF is started	386	376	406	383	394	384
$Y_{\mathfrak{a}_1(1),\mathfrak{a}_3(1)}$	intervention, BF is started	421	429	406	422	430	437
$Y_{\mathfrak{a}_2(1),\mathfrak{a}_3(1)}$	programme followed, BF is started	448	429	482	437	463	437
$Y_{\mathfrak{a}_4(1)}$	duration BF = 3 months	522	493	576	501	546	508

'prog' are women who followed the breastfeeding programme ($A_2^{obs} = 1$)

'noprog' are women who received an invitation but did not follow the breastfeeding programme ($A_2^{obs} = 0$ and $A_1^{obs} = 1$)

BF.interv are women who started breastfeeding in the intervention group ($A_3 = 1$ and $A_1 = 1$)

no BF are women who did not start breastfeeding in the control group ($A_3 = 0$ and $A_1 = 1$) Compliers are women who will start breastfeeding if programme is offered, and not, when it is not offered ($A_{3,\mathfrak{a}_1(1)} = 1$ and $A_{3,\mathfrak{a}_1(0)} = 0$)

6.1 Causal effects in the total population

The true causal effect which would be calculated had this been a randomized clinical trial (intention-to-treat effect), would be $E[Y_{\mathfrak{a}_1(1)} - Y_{\mathfrak{a}_1(0)}] = 99$ grams. If everyone were to ac-

tually follow the programme the difference would be $E[Y_{\mathfrak{a}_2(1)} - Y_{\mathfrak{a}_1(0)}]$= 164 grams. If the programme were offered to everyone and everyone started breastfeeding, the difference, relative to no programme, would be $E[Y_{\mathfrak{a}_1(1),\mathfrak{a}_3(1)} - Y_{\mathfrak{a}_1(0)}]$ =234 grams. If everyone were to follow the programme and all started breastfeeding this would be $E[Y_{\mathfrak{a}_2(1),\mathfrak{a}_3(1)} - Y_{\mathfrak{a}_1(0)}]$ =261 grams, a slightly larger effect because the programme increases the mean duration of breastfeeding. Had everybody started breastfeeding, without following the programme, the increase in weight would be $E[Y_{\mathfrak{a}_1(0),\mathfrak{a}_3(1)} - Y_{\mathfrak{a}_1(0)}]$ =199 grams. The difference compared to the situation where no one would start breastfeeding is $E[Y_{\mathfrak{a}_1(1),\mathfrak{a}_3(1)} - Y_{\mathfrak{a}_3(0)}] = 421$ grams.

Some of the causal effects described above are not very realistic. Not every woman would be able to start breastfeeding. For example when a mother becomes very ill at the end of pregnancy, breastfeeding her baby may not be an option because of toxicity of prescribed medication or poor health. Assuming that every woman would continue breastfeeding for 3 months is even more unlikely.

This shows that some of the causal effects which may be estimable are unrealistic large. In our example the largest causal contrast is the expected weight difference when every infant versus none is breastfed for 3 months, which is equal to $E[Y_{\mathfrak{a}_4(1)} - Y_{\mathfrak{a}_3(0)}]$= 522 grams.

6.2 Causal effects in sub-populations

In our example the "average treatment effect in the treated (ATT)" can be defined in different ways. "Treated" could mean actually following the programme, if offered. In this case the effect of attending the programme is ATT $= E[Y_{\mathfrak{a}_2(1)} - Y_{\mathfrak{a}_1(0)}|A_2 = 1]$= 153 grams. The corresponding Average Treatment effect among the non Treated then is ATNT $= E[Y_{\mathfrak{a}_2(1)} - Y_{\mathfrak{a}_1(0)}|A_2 = 0$ and $A_1 = 1]$= 180 grams. Alternatively, treated could mean being breastfed in which case the ATT is the effect of breastfeeding in those who actually start breastfeeding: $E[Y_{\mathfrak{a}_3(1),\mathfrak{a}_1(1)} - Y_{\mathfrak{a}_3(0)}|A_3 = 1] = 417$ grams and the ATNT is $E[Y_{\mathfrak{a}_3(1),\mathfrak{a}_1(1)} - Y_{\mathfrak{a}_3(0)}|A_3 = 0] =$ 426 grams.

Another local effect which may be of interest is the CACE, the Complier Average Causal Effect. The CACE for the BEP intervention is CACE= $E[Y_{\mathfrak{a}_1(1)}-Y_{\mathfrak{a}_1(0)}|A_{3,\mathfrak{a}_1(1)} = 1$ and $A_{3,\mathfrak{a}_1(0)} = 0]$ =437 grams. In our study, the CACE represents the effect of the programme in the subgroup of individuals whose decision to start breastfeeding depends on allocation to the BEP programme.

When implementing an intervention, it is of interest to identify those subgroups for which the intervention is most beneficial. Table 6 shows for example that infants of less educated women will profit more than those of more educated women, both when the programme is offered and when the programme is actually followed.

Table 6: Potential weight differences under different scenarios compared to no BEP programme, in sub-populations, calculated from data generated as in PROBITsim Study, but with $N = 5,000,000$.

		Education			Smoking	
Outcome	Scenario	low	medium	high	yes	no
$Y_{\mathfrak{a}_3(0)}$	No BF	0	0	0	0	0
$Y_{\mathfrak{a}_1(0)}$	intervention is not offered	185	198	154	181	190
$Y_{\mathfrak{a}_1(1)}$	intervention is offered	296	297	223	279	289
$Y_{\mathfrak{a}_2(1)}$	programme is followed	395	351	242	401	333
$Y_{\mathfrak{a}_1(0),\mathfrak{a}_3(1)}$	no intervention, BF is started	425	393	262	470	355
$Y_{\mathfrak{a}_1(1),\mathfrak{a}_3(1)}$	intervention, BF is started	477	420	281	507	389
$Y_{\mathfrak{a}_2(1),\mathfrak{a}_3(1)}$	programme followed, BF is started	528	436	285	564	405
$Y_{\mathfrak{a}_4(1)}$	duration BF = 3 months	663	483	302	680	463

7 Variables in the dataset

Our simulated dataset of 17044 women contains the following variables:

A1	Randomisation; A1=1 is intervention, A1=0 is control
A2.observed	Followed the intervention programme (1=yes, 0 = no)
Location	location of living. (1: urban western region, 2: rural western region, 3: urban, eastern region and 4: rural, eastern region)
Age	Age of women at randomisation
Educ	Education level 1=low, 2=medium, 3=high
Smoke	Smoking during pregnancy (1=yes, 0 = no)
Allergy	Maternal allergy. (1=yes, 0 = no)
Caesarean	Child born by Caesarian (1=yes, 0 = no)
Wgt0	Birth weight in grams
Sex	Sex of child, (1=boy, 0 = girl)
A3.observed	Breastfeeding started (1=yes, 0 = no)
dur.observed	Duration of breastfeeding in the first three months
A4.observed	Completed 3 months of breastfeeding(1=yes, 0 = no)
Wgt3.observed	Weight of child after 3 months
A2potential	The women follows the programme if it is offered (yes/no)
A3pot.A1.0	The women will start breastfeeding, if no intervention is given (yes/no)
A3pot.A1.1	The women will start breastfeeding, if intervention is given (yes/no)
A3pot.A1.1.A2.1.	The women will start breastfeeding, after following the programme (yes/no)
durpot.A1.0	Potential duration of breastfeeding, under no intervention
durpot.A1.1	Potential duration of breastfeeding, under intervention
durpot.A1.1.A2.1	Potential duration of breastfeeding, when programme is followed
durpot.A1.0.A3.1	Potential duration of breastfeeding, under no intervention, but breastfeeding is started
durpot.A2.1.A3.1	Potential duration of breastfeeding, when programme is followed and breastfeeding is started
durpot.A2pot.A3.1	Potential duration of breastfeeding, when intervention is given and breastfeeding is started
Wgt3pot.A1.0	Potential weight at 3 months under no intervention
Wgt3pot.A1.1	Potential weight at 3 months under intervention
Wgt3pot.A2.1	Potential weight at 3 months after following programme
Wgt3pot.A1.0.A3.1	Potential weight at 3 months under no intervention but breastfeeding is started.
Wgt3pot.A1.1.A3.1	Potential weight at 3 months under intervention and breastfeeding is started
Wgt3pot.A2.1.A3.1	Potential weight at 3 months when programme is followed and breastfeeding is started
Wgt3pot.dur0	Potential weight at 3 months when breastfeeding duration = 0 months
Wgt3pot.dur1	Potential weight at 3 months when breastfeeding duration = 1 months
Wgt3pot.dur2	Potential weight at 3 months when breastfeeding duration = 2 months
Wgt3pot.dur3	Potential weight at 3 months when breastfeeding duration = 3 months

Figure 2: Data generating model for A_2 and A_3 in terms of A_1, $\boldsymbol{L}_1$ and $\boldsymbol{L}_2$

.

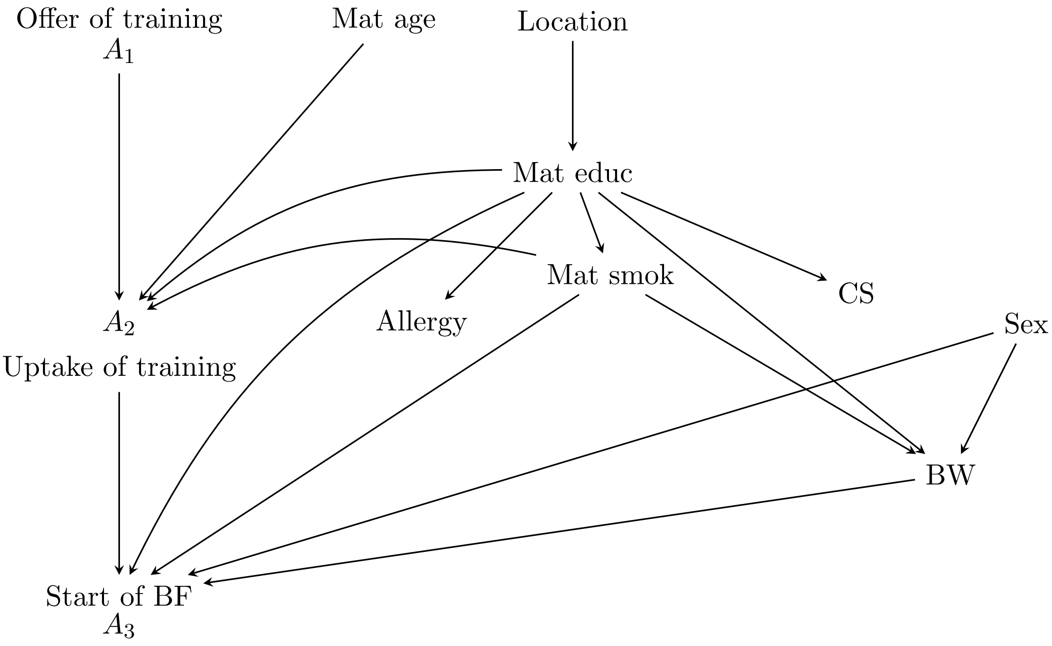

BF: breastfeeding; BW: birth weight; CS: caesarian section;

$\boldsymbol{L}_1$: maternal age, education, smoking during pregnancy; $\boldsymbol{L}_2$: birth weight and sex of the baby

Figure 3: Data generating model for A_4 in terms of A_1, $\boldsymbol{L}_1$ and $\boldsymbol{L}_2$

†.

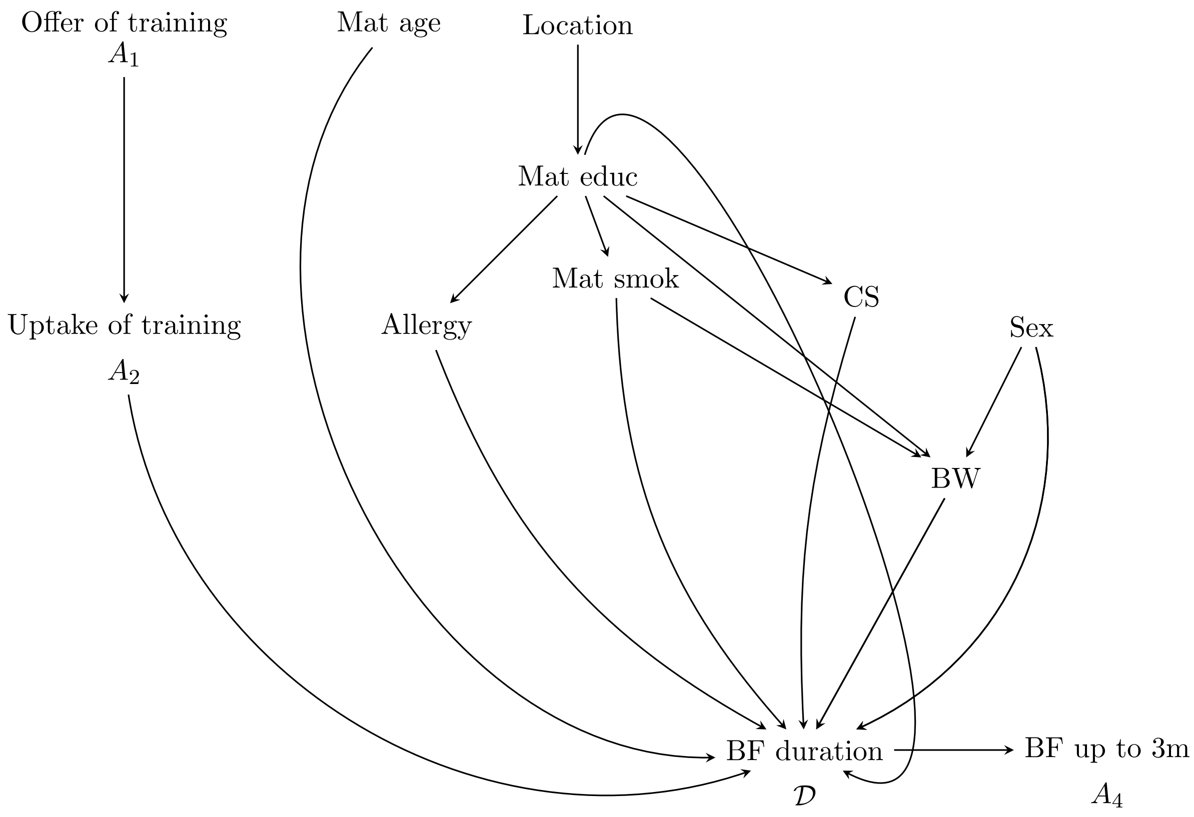

† A_3 is not depicted as it is part of $\mathcal{D}$

BF: breastfeeding; BW: birth weight; CS: caesarian section

$\boldsymbol{L}_1$: maternal age, education, smoking during pregnancy; $\boldsymbol{L}_2$: birth weight and sex of the baby

Figure 4: Data generating model for infant weight at 3 months in terms of A_1, A_2, $\boldsymbol{L}_1$ and $\boldsymbol{L}_2$ †

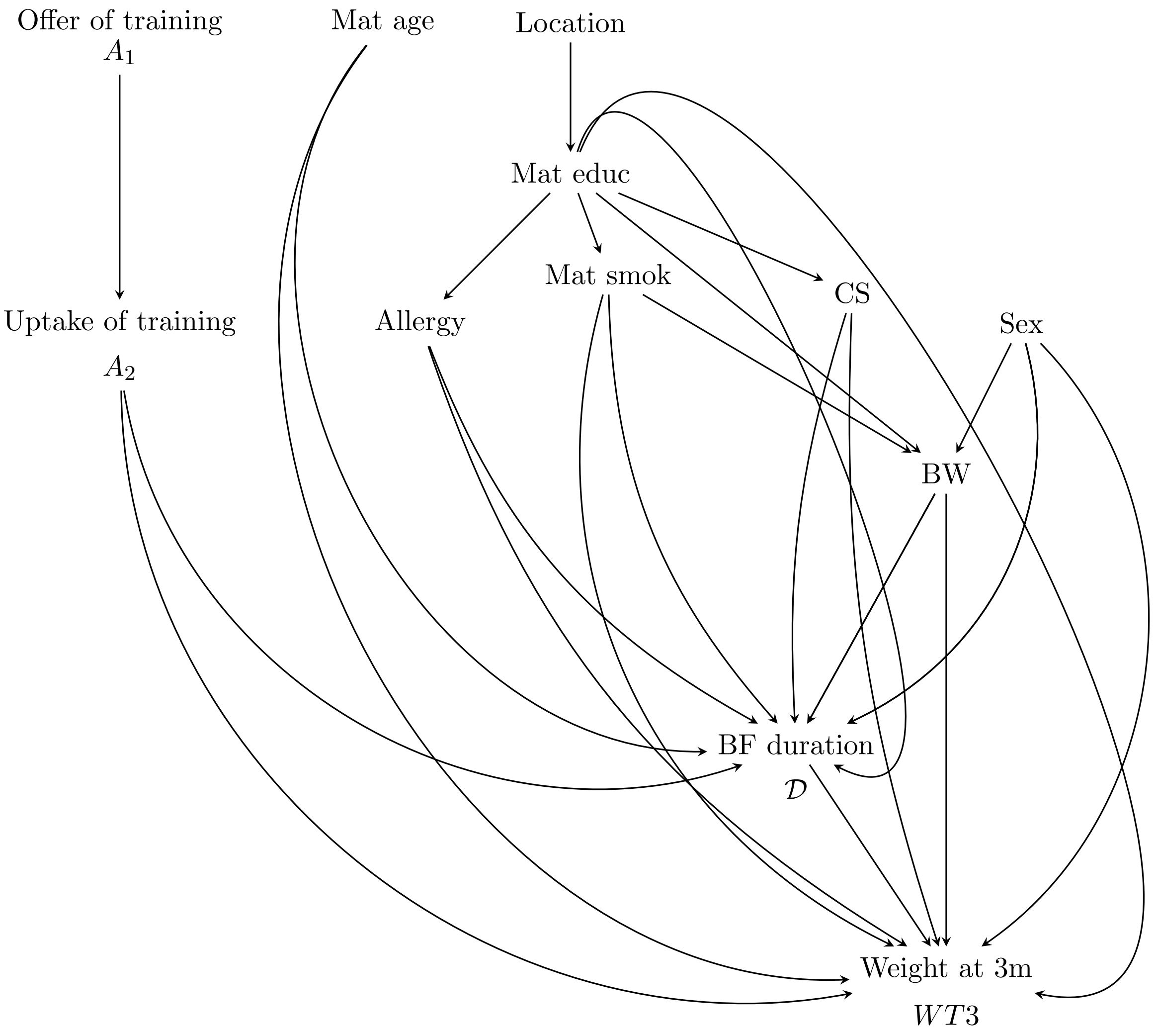

† A_3 is not depicted as it is part of $\mathcal{D}$

BF: breastfeeding; BW: birth weight; CS: caesarian section

$\boldsymbol{L}_1$: maternal age, education, smoking during pregnancy; $\boldsymbol{L}_2$: birth weight and sex of the baby
