# Supplementary material for: Formulating causal questions and principled statistical answers
Source: Stat Med. 2020 Sep 23;39(30):4922–48. doi: 10.1002/sim.8741 (PMC7756489; doi:10.1002/sim.8741)
Supplement: Supplementary file 2 — Data S2. Appendix 2 [file SIM-39-4922-s002.pdf]

Appendix 2 — Analyses of the *simulation learner* PROBITsim

1 Background

The generation of PROBITsim is described in Appendix 1. Briefly, the data are inspired by the Promotion of Breastfeeding Intervention Trial (PROBIT) (Kramer et al, 2001) in which mother-infant pairs across 31 Belarusian maternity hospitals were cluster randomised to receive either standard care or a breastfeeding encouragement intervention to investigate the effect of breastfeeding on a child's later development. The aim of the simulated data set, with individual randomisation of mother-infant pairs, together with these analyses is to provide a practical example that can be used to implement the estimation approaches described in the paper. The results reported in this document were obtained using Stata and are described as if they pertained to a real trial. Equivalent analyses were produced using R and SAS. Code specific to each package and results for the latter two can be found on the website `ofcaus.org` and on `https://github.com/IngWae/Formulating-causal-questions`. Differences in estimates and confidence intervals found across softwares are due to computational variations (including bootstrap implementations).

2 Data description

Participants

The simulated dataset holds records on 17,044 records. All variables have complete information. The distribution of these variables was similar across assigned intervention groups (Table 1). A total of 8,667 (50.9%) pregnant women were assigned to the intervention arm, and 8,377 (49.1%) to the control arm.

Mean maternal age at birth was 24.3 years and 27.3% of mothers smoked during pregnancy. There was a majority of baby boys (51.8%) and the overall mean birth weight was 3028g. The distribution of the outcome of interest, weight at 3 months, was fairly symmetrical, with mean 6064g and SD=594g (Table 1).

Average birth weight was lower in girls (girls' mean birth weight 2961g), and in babies whose mothers had lower education (2944g); average birth weight was lower also for babies whose mother smoked during pregnancy (2871g).

Uptake of the programme

The uptake of the Breastfeeding Encouragement Programme (BEP) among those assigned to the intervention group (i.e. those for whom $A_1 = A_2 = 1$) was 64.4%. Among those assigned to the programme, uptake of breastfeeding was greater than among those not assigned to the programme (67.9% vs 49.5%; Table 2, top). Furthermore, overall, among those that attended the programme, more started breastfeeding than among those that were invited but did not attend it (81.4% vs. 43.4%; Table 2, bottom).

Table 1: Mean values of the main characteristics of the study participants by treatment arm (A_1) and within arm, by observed values of selected combinations of A_2, A_3 and A_4; Simulation Learner PROBITsim Study: N=17,044

		Intervention group						Control group			
			$A_1 = 1$	$A_1 = 1$	$A_1 = 1$	$A_1 = 1$	$A_1 = 1$		$A_1 = 0$	$A_1 = 0$	$A_1 = 0$
Variable	Overall	$A_1 = 1$	$A_2 = 1$	$A_2 = 0$	$A_3 = 1$	$A_3 = 0$	$A_4 = 1$	$A_1 = 0$	$A_3 = 1$	$A_3 = 0$	$A_4 = 1$
Location= 1	0.33	0.32	0.33	0.31	0.33	0.31	0.33	0.34	0.34	0.34	0.35
Location= 2	0.16	0.17	0.16	0.17	0.17	0.17	0.16	0.16	0.17	0.15	0.17
Location= 3	0.26	0.26	0.26	0.26	0.27	0.25	0.27	0.27	0.26	0.27	0.26
Location= 4	0.24	0.25	0.24	0.26	0.24	0.28	0.24	0.23	0.23	0.24	0.22
Mother's age (y)	24.3	24.3	24.9	23.1	25	22.7	25.3	24.3	25.3	23.2	25.6
Educ= low	0.36	0.36	0.30	0.48	0.32	0.45	0.25	0.35	0.31	0.39	0.21
Educ= medium	0.50	0.5	0.53	0.45	0.52	0.46	0.56	0.51	0.52	0.5	0.57
Educ= high	0.14	0.14	0.17	0.07	0.16	0.09	0.19	0.14	0.17	0.11	0.21
Maternal smoking	0.27	0.27	0.19	0.43	0.21	0.40	0.18	0.27	0.20	0.34	0.18
Allergy	0.04	0.04	0.05	0.04	0.04	0.04	0.05	0.05	0.05	0.04	0.06
Caesarean birth	0.12	0.12	0.12	0.11	0.12	0.11	0.1	0.12	0.13	0.12	0.09
Male infant	0.52	0.52	0.52	0.52	0.54	0.48	0.52	0.51	0.52	0.51	0.5
Birth weight (g)	3028	3024	3049	2979	3070	2927	3111	3032	3099	2967	3158
Weight 3 mths (g)	6064	6110	6196	5955	6297	5715	6388	6016	6270	5767	6407

Numbers are means or proportion of individuals with the characteristic

3 The effect of randomization into the programme (A_1)

In this section we address the question *"What would the average infant weight be at 3 months, had all mothers been offered the programme, versus had all mothers not been offered the programme?"*. The corresponding estimand is the ATE of A_1. Because the exposure (being offered the programme) is randomised, this causal effect is also an intention-to-treat (ITT) effect.

To estimate this effect on a mean difference scale we simply fit a linear regression model of weight at 3 months with A_1 as the only explanatory variable or, equivalently, we calculate the difference between the observed means in those assigned to the programme and those not assigned to the BEP. This estimate (95% confidence interval) is 94.2g (76.4,112.0) and it indicates that inviting expecting mothers to attend this specific programme would increase their baby's birth weight by 94.2g, on average.

4 The effect of uptake of the Breastfeeding Encouragement Programme (A_2)

Here we wish to address the question *"What would the average infant weight be at 3 months, had all mothers been offered the BEP and all had followed it, versus had all mothers not been offered the programme?"*. The corresponding estimand is the ATE of A_2.

Another question could be *"What would the difference in average infant weight at 3 months be, had all mothers who had attended the BEP when offered, not attended it?"*. The corresponding estimand is the ATT of A_2.

Since uptake of the programme is not randomised, the estimation of this causal effect on infant weight at 3 months is not straightforward. If we adopt any of the estimation methods that require the NUC assumption we need to identify a sufficient set of confounders that would remove any non-causal associations between A_2 and infant weight at 3 months. If we adopt an estimation method that requires instrumental variables, then we need instead to identify one such variable and to measure it.

Table 2: Frequency distribution of study participants by the three main treatments of interest.

Overall		
	$A_1 = 0$ N (%)	$A_1 = 1$ N (%)
$A_2 = 0$	8377 (100)	3083 (35.6)
$A_2 = 1$	0 (0)	5584 (64.4)
$A_3 = 0$	4226 (50.5)	2782 (32.1)
$A_3 = 1$	4151 (49.5)	5885 (67.9)
All	8377 (100)	8667 (100)

Among those with $A_1 = 1$		
	$A_2 = 0$ N (%)	$A_2 = 1$ N (%)
$A_3 = 0$	1745 (56.6)	1037 (18.6)
$A_3 = 1$	1338 (43.4)	4547 (81.4)
All	3083 (100)	5584 (100)

A_1: Offer of BEP;
A_2: Uptake of BEP;
A_3: Start of breastfeeding.

In either case we also will invoke the assumptions of no interference and causal consistency. In this context these assumptions mean the following:

- No interference means that the uptake of the BEP by one mother does not influence the weight at 3 months of the infant of another mother. This is plausible if mothers in the study do not share the knowledge acquired if/when attending the programme.
- Consistency means that the BEP is well defined in the sense that the hypothetical weight of the infant had their mother attended/not attended the programme is the same as the observed weight when the mother actually attended/did not attend the programme. This is plausible if attending the programme as opposed to forcing attendance has the same impact on the outcome.

4.1 Estimation approaches requiring the NUC assumption

The NUC assumption is met if there is a set of measured covariates that would remove any non-causal associations between A_2 and infant weight, if controlled for. In our example this set is $\boldsymbol{L}_1$ in Figure 1, Appendix 1, with details of how the variables in $\boldsymbol{L}_1$ relate to the other variables involved in the data generation of the outcome shown in Figures 2-4 in Appendix 1. These figures show that a minimal set of confounders that would meet the NUC assumption includes: maternal age and educational level before the birth of the child and smoking status during pregnancy. However, since the true data generating structure would not be known to us, we use a larger adjustment set in the analyses, as described below.

Each of the estimation methods that invoke the NUC assumption (as well as no interference and causal consistency), also requires some additional assumptions that will be outlined when we describe their implementation. Although we report in Table 3 the estimates of ATE_2 and ATT_2 obtained by each method , we focus on interpreting and comparing estimates of ATE_2

in the text.

4.1.1 Outcome regression

Outcome regression involves selecting a parametric model for the outcome that involves the exposure and the confounders. We demonstrate three regression approaches. First we use a crude (unadjusted) regression which fits a linear regression model of weight at 3 months with BEP uptake A_2 as the only explanatory variable. The resulting estimate, identical to the mean difference of observed outcomes between treatment groups, is 196.0g (177.2,214.8); see Table 3. In a second model we add the covariates maternal education, smoking during pregnancy, history of allergy, location of living, age (centered) and the square of the centered age variable. This model gives an estimated ATE for A_2 of 155.4g (136.8,174.0). An alternative specification of the model includes interactions between A_2 and each of the confounders. This yields $\widehat{\text{ATE}}_2$= 165.0g (146.0,184.0).

Interpreting either of these adjusted estimates as causal requires the assumption that their respective models are correctly specified, in addition to the NUC assumption. As the latter model is more general, $\widehat{\text{ATE}}_2 = 165.0$g is the more plausible.

4.1.2 Propensity score based methods

Instead of modelling the outcome, we could model the association between confounders and exposure.

Such model for the propensity score (PS), is used to estimate covariate-specific probabilities of being exposed. These propensities are then used in different ways: as a stratifying variable (PS stratification); as a single additional covariate in the outcome model (Regression with PS); as a matching variable (PS matching); as a weight (IPW).

For the methods to lead to causal effect estimates, in addition to no interference, consistency and NUC, positivity and correct specification of the propensity score model need to be assumed. Positivity can be checked visually by plotting the distribution of the predicted propensity scores separately by exposure status.

Using the same confounders as in 4.1.1, and also allowing for non-linearities, we specify the logistic regression model for exposure A_2 as:

$$\text{logit}[\Pr(A_2|\boldsymbol{L})] = \alpha + \boldsymbol{\beta}^T\boldsymbol{L}$$

where $\text{logit}(p) = \log\frac{p}{(1-p)}$ and $\boldsymbol{L}$ includes the same presumed confounders as before (i.e. maternal age and education, smoking during pregnancy, history of allergy, and location of living). The square of (centered) age was also included in the vector $\boldsymbol{L}$ to specify the PS model as generally as possible.

Figure 1 shows the distribution of the fitted values of this propensity score (PS) model separately by observed values of A_2. The figure indicates good overlap in the propensity of being exposed between the two groups, hence supporting the assumption of positivity.

Using this PS to re-weight the observations by the inverse of the probability of being treated with the observed treatment leads to a rebalancing of the covariates across exposure groups, as shown in Table 4. In particular the original imbalances in education, smoking and age across exposure groups are not evident anymore after re-weighting.

Stratification

An approach for confounder adjustment based on the propensity score involves performing a stratified analysis. Here, individuals with similar propensity scores are grouped together in

strata and within each strata the mean difference in outcomes is calculated. Often quantiles of the propensity score distribution are used to define the strata. We use a PS stratification estimator where the six strata are defined by the sextiles of the fitted propensity score distribution. A key assumption underlying the analysis is that, within each stratum, there is no further variation in the PS. The ATE, estimated by the mean of the stratum-specific differences with bootstrap standard error, is 165.0g (146.6,183.4); see Table 3.

Regression adjustment with the PS

An alternative approach is to include the fitted PS in the outcome model in place of the confounders. To achieve this we fitted a linear regression model with weight at three months as the outcome and treatment A_2 together with the PS as the covariates and obtained an estimated ATE of 156.2g (138.6,173.8); see Table 3. In order for the regression adjustment with the PS to be a consistent estimator of ATE_2 we need that i) the propensity score model is correctly specified and ii) the outcome regression model is either correctly specified or linear with a homogeneous effect conditional on the PS (in addition to the assumptions of no interference, consistency, NUC and positivity).

PS Matching

A matching estimator matches each unit to one or several units from the opposite treatment group. Matching with more than one unit may lead to bias if the matching is not satisfactory but reduces the variance and thereby the mean squared error. Hence, here we demonstrate both 1:1 matching and 1:3 matching. The matching is done with replacement, meaning that each individual can be used as a match more than once. In Table 3 the ATE_2 estimated with 1:1 matching is 155.7g (135.9,175.5) and that with 1:3 matching is 154.9g (135.1,174.7), where the standard errors are computed according to Abadie and Imbens (2012). We note that the standard errors and confidence intervals for both matching schemes are somewhat wider than for the all other NUC approaches reviewed so far.

The matching estimator requires that the matching criterion (here the estimated PS) converges to a balancing score, that is,

$$A_2 \perp\!\!\!\perp L \mid \hat{e}(L), \tag{1}$$

i.e, that the estimated PS captures the information held in the covariates that predict A_2, i.e. the uptake of the programme. This is a larger class of functions than the true propensity score $e(\boldsymbol{L})$, thus adopting this estimation approach calls upon a slightly milder assumption than the assumption that the PS model is correctly specified. The balance after matching is assessed in Figure 2.

Inverse probability weighting

An inverse probability weighting (IPW) estimator is formed by weighting each observed outcome by the inverse of the PS, i.e., the probability of the treatment received conditional on the covariates, thereby accounting for the confounding because re-weighting by the PS removes the association between the exposure and the confounders. The mean difference of the weighted outcomes is then used as an estimator of the ATE. We use the normalized version of the IPW-estimator, which gives an estimate for ATE_2 of 164.7g (146.5,182.9); see Table 3. The IPW-estimator is consistent if the PS model is correctly specified.

Augmented inverse probability weighting

The augmented inverse probability estimator (AIPW), sometimes also referred to as a doubly robust (DR) estimator requires the specification of both the PS model and the outcome model. Here, we use the outcome model described in 4.1.1 which includes the interactions between A_2 and all the confounders. For the AIPW estimator we assume that at least one of the models (i.e. PS or outcome model) is correctly specified. The estimated ATE for A_2 is 164.7g (145.7,183.7); see Table 3.

4.2 IV-based estimation approaches

In this setting randomization to the breastfeeding programme (A_1) can be viewed as an instrumental variable for the relationship between A_2, programme uptake, and infant weight at 3 months. Indeed the three core assumptions for A_1 to be an IV are met as can be seen from Figure 1, Appendix 1:

- IV1 A_1 is associated with the uptake of the programme
- IV2 A_1 is independent of the confounders $\boldsymbol{L}_1$ of the relationship between uptake and infant weight
- IV3 A_1 is independent of infant weight, conditionally on A_2 and the confounders $\boldsymbol{L}_1$.

Hence we can use A_1 as an instrument to test whether A_2 has a causal effect on infant weight at 3 months and to estimate the causal effect of A_2, without requiring the NUC assumption but invoking these alternative assumptions instead.

A test for a causal effect of A_2 on weight at 3 months can be carried out by assessing the significance of the ITT effect of A_1, i.e. the significance of the instrument. We have already indirectly seen this to be case in Section 3 where we estimated the causal effect of A_1 and found its 95% CI not to include 0.

Adopting IV estimation leads to an estimate of 146g (119,174). This can be interpreted as the ATE of A_2 if we additionally assume that the effect of programme uptake is the same for all infants. An alternative weaker assumption that would lead to the same interpretation is that there is no effect modification on the additive scale between the instrument A_1 and the exposure A_2. This may not be realistic however, while the same assumption restricted to the exposed leads to the IV estimate to be interpreted as an ATT. This approach gives the largest standard error which implies that the 95% CI is much wider than those obtained using any of the estimation approaches relying on the NUC assumption.

Note that the IV analysis described above can be repeated adjusting for known confounders and this may decrease the width of the confidence interval.

5 The effect of uptake of breastfeeding (A_3) among those offered ($A_1 = 1$) and among those not offered ($A_1 = 0$) the programme

We might wish to address the question *"What would the infant weight at 3 months be, had all mothers started breastfeeding, versus had they not started at all?"*. We study the question in two alternative scenarios: i) In the world without the offer of the BEP ($A_1 = 0$) and, ii) in the world where mothers have been offered the programme ($A_1 = 1$). The corresponding estimands are the ATEs in the two strata defined by A_1.

Since not all mothers may be able to breastfeed or be advised not to breastfeed for medical reasons, the usefulness of this estimand could be questioned. An alternative question could instead be: *"What would the difference in infant weight at 3 months be, had all mothers who started breastfeeding not started at all?"*. Formally this is a causal question restricted to the treated, i.e. an ATT. For those starting to breastfeed, i.e. those for whom $A_3 = 1$, we can no longer use A_1 as instrument because A_1 is not independent of the outcome conditional on A_3 (i.e. IV3 is not met): A_1 increases the odds of starting breastfeeding and the duration of breastfeeding, and hence infant weight at 3 months via pathways that do not involve A_3 (Figure 1, Appendix 1). For this reason we use here only estimation methods that invoke the NUC assumption.

Again we will assume no interference and consistency. In this context these mean the following:

- The assumption of no interference means that we are expecting that the uptake of breastfeeding by one mother does not influence the weight at 3 months of other infants.

- Consistency means that the start of breastfeeding is well defined in the sense that the hypothetical weight of the infant had their mother started to breastfeed is the same as the observed weight for the infants of mothers who actually started to breastfeed. This is plausible if self-imposed breastfeeding as opposed to starting breastfeeding directed by an intervention has the same impact on the infant's weight at 3 months.

5.1 NUC estimation approaches

To estimate either of these effects we again call upon the NUC assumption, in addition to assuming no interference and consistency. Assuming NUC requires identifying a sufficient set of confounders of the causal effect of A_3 on infant weight at 3 months. These are covariates that, when controlled/adjusted for, block all non-causal paths between A_3 on infant weight at 3 months. Examining Figures 2–4 in Appendix 1 we identify the following:

- Among the $\boldsymbol{L}_1$ variables: maternal age, education, smoking during pregnancy.

- Among the $\boldsymbol{L}_2$ variables: birth weight and sex of the baby.

- Among the treatments A_2, but only when examining the population defined by $A_1 = 1$ (because when $A_1 = 0$ then $A_2 = 0$ by design).

In practice however we would not have information on the true data generating structure and therefore below we add to the set of confounders birth by caesarean section and maternal allergy.

In the following we report estimates of both ATE and ATT in the two subpopulations defined by A_1.

5.1.1 Outcome regression

To adopt this approach we require that the additional assumption that the outcome model is correctly specified. For this reason we include quadratic terms for maternal age and birth weight (after centering these variables around their means) and also interactions between A_3 and each of the confounders. This led to estimating an ATE 384.7g (378.4,391.0) in the stratum $A_1 = 0$ and an ATE of 425.3 (420.0,430.6) in the stratum $A_1 = 1$ (Table 5).

Note that the observed mean weight difference between breastfed and not breastfed infants is of 503.2g (480.5,525.9) in the stratum $A_1 = 0$ and 582.0g (558.1,605.9) in the stratum $A_1 = 1$.

5.1.2 Propensity score based methods

The assumptions required here (in addition to no interference, consistency, NUC) are positivity and correct specification of the PS model. Figures 3 and 4 show these distributions in the two subpopulations defined by A_1 and indicate good overlap in propensity of exposure between the groups defined by A_3.

We fitted two PS models, one per subpopulation, using logistic regression with the covariates: maternal education, smoking status during pregnancy, age (centered), (centered) age squared, allergy, location of living, birth by caesarean section, sex, birth weight (centered) and (centered) birth weight squared. The inclusion of A_2 as a covariate was only made when fitting the PS model in the subpopulation defined by $A_1 = 1$.

Re-weighting the observations by the inverse of the PS fitted in the two populations leads again to a rebalancing of the covariates across exposure groups (Table 6).

Stratification

For each subpopulation (defined by A_1) we used six strata where the sample was divided into sextiles defined by the distribution of the PS estimated in that subpopulation. The estimate of the ATE was then obtained by averaging the stratum-specific mean differences. For infants in the $A_1 = 0$ subpopulation the estimate was 392.2g (384.2,400.2) and 442.0g (429.3,454.7) for those in the $A_1 = 1$ subpopulation; see Table 2. To estimate the ATT we defined instead the six strata by dividing the treated (i.e. those with $A_3 = 1$) into six groups of equal size and then taking the average of the stratum-specific differences. This gave an estimated ATT of 388.8g (379.4,398.2) in the subpopulation with $A_1 = 0$ and 438.3g (419.7,456.9) in that with $A_1 = 1$ (Table 5).

Regression adjustment with the PS

Fitting an outcome model with the uptake of breastfeeding A_3 and the fitted PS as the only predictors gives an estimate of the ATE of 384.4g (378.1,390.7) in the subpopulation with $A_1 = 0$ and 425.9g (419.4,432.4) in that with $A_1 = 1$ (Table 5).

Matching

For the matching estimator we assume that the fitted propensity scores are balancing scores as described in Equation 1. For 1:1 matching we get estimates of the ATE of 386.5g (359.6,413.3) in the subpopulation with $A_1 = 0$ and 429.0g (394.9,463.1) in the subpopulation with $A_1 = 1$. Balance plots for mean differences in each covariate (before and after matching) are displayed in Figures 5 and 6 for evaluating the matching balance. For 1:3 matching the standard error is smaller and the estimated ATEs are 380.7g (356.4,405.0) and 437.2g (407.4,467.0) respectively (Table 5).

Inverse probability weighting

Using the inverse of the fitted PS as a weight to control for confounding we assume that the PS model is correctly specified in each of the subpopulations. This approach leads to an estimated ATE of 384.7g (377.3,392.1) in the subpopulation with $A_1 = 0$ and 426.6g (412.7,440.5) in the subpopulation with $A_1 = 1$ (Table 5).

Augmented inverse probability weighting

The augmented inverse probability estimator relies on the assumption that at least one of the PS or outcome regression models is correctly specified. Here the estimated ATEs are 384.8g (377.0,392.6) and 426.7g (412.4,441.0), respectively (Table 5).

6 The effect of A_3 among low educated mothers that were offered the breastfeeding programme ($A_1 = 1$)

Here, we study the subpopulation of infants of mothers with low education who were offered the breastfeeding programme. In this subpopulation we address the question *"What would the infant weight at 3 months be, had all mothers with low level of education and who were offered the BEP started breastfeeding, versus the infant weight had they not started at all?"*. This is a conditional ATE (conditional on one level of education).

6.1 NUC estimation approaches

To estimate this effect we again call upon the NUC assumption, in addition to assuming no interference and consistency. Since the subpopulation is selected by education and randomization status, a sufficient set of variables to be adjusted/controlled for includes maternal age, smoking, birth weight, sex of the baby, and uptake of the programme (A_2). However, in the analyses below, as before, we pretend not to know the data generating structure and therefore we additionally control for: location of living, birth by caesarean section and maternal allergy status.

6.1.1 Outcome regression

The unadjusted mean weight difference in the two exposure groups is 626.1g (587.3,664.9). Fitting an outcome regression model with all the confounders (and squared terms for maternal age and birth weight) but no interactions with A_3 gave an estimate of 471.6g (458.9,484.3). Including these interactions gave an estimated ATE of A_3 for babies of low educated mothers of 481.0g (469.6,492.4) see Table 7. To interpret any of these estimates as causal we invoke the assumption that the outcome model is correctly specified, hence the latter estimate is the most reliable as based on the more general specification of the model.

6.1.2 Propensity score based methods

For the PS-based methods we rely on the correct specification of the PS model. Here we use the same logistic regression model as in Section 5 that concerned women that were offered the programme (hence A_2 was included in the PS model).

6.2 Regression adjustment with the PS

Using a regression model with the exposure A_3 and the fitted PS as explanatory variables we can estimate the ATE for A_3 under the assumption that the linear regression model is correctly specified or that the effect of A_3 does not vary with the PS. This approach results in an estimate of the ATE of A_3 for babies of low educated mothers of 470.9g (458.2,483.6); see Table 7.

Stratification

A stratification estimator using six strata based on the fitted PS results in an estimate of the ATE of A_3 for infants of low educated mothers of 497.7g (476.7,518.7); see Table 7. Using this approach we assume that the PS model is correctly specified and that there is no residual imbalance within the strata.

Matching

The matching approach assumes that the PS is a balancing score. For 1:1 matching the ATE was estimated to be 482.3g (429.6,535.0); see Table 7. For 1:3 matching the ATE estimate was 472.6g with a slightly smaller variance than the 1:1 matching yielding a 95% confidence interval of 425.6 to 519.6g. Here, the standard errors for both the 1:1 and 1:3 matching estimators are much larger than for the other NUC-based estimators (Table 7).

Inverse probability weighting

Using inverse probability weighting the estimate of the ATE of A_3 for babies of low educated mothers was estimated to be 482.7g (461.1,504.2); see Table 7.

Here, again, the additional assumption is that the PS is correctly specified.

Augmented inverse probability weighting

Adopting this approach the ATE was estimated to be 482.7g (461.9,503.0); see Table 7. For this to be interpreted causally we assume that either the PS or the outcome regression model is correctly specified.

Table 3: Estimated ATE and ATT of A_2 on weight at 3 months (in grams) obtained using alternative estimation methods controlling for relevant confounders*; PROBITsim Study.

Estimand	Estimation method	Estimate	(SE)
ATE			
	True value	165.1	
	Crude regression	196.0	(9.6)
	Regression adjustment (without interactions)	155.4	(9.5)
	Regression adjustment (with interactions)	165.0	(9.7)
	PS stratification† (6 strata)	165.0	(9.4)
	Regression with PS †	156.2	(9.0)
	PS matching (1 match)‡	155.7	(10.1)
	PS matching (3 matches)‡	154.9	(10.1)
	PS IPW†	164.7	(9.3)
	PS DR IPW†	164.7	(9.7)
	IV	146.2	(14.0)
ATT			
	True value	152.8	
	Regression adjustment (with interactions)	148.7	(9.4)
	PS stratification† (6 strata)	148.7	(9.6)
	PS matching (1 match)‡	145.8	(9.8)
	PS matching (3 matches)‡	145.4	(9.7)
	PS IPW†	148.0	(9.6)

* The variables controlled for in each of these analyses were: maternal age (linear and quadratic term), maternal education, maternal allergy status, smoking status in the first trimester (i.e. before programme allocation), and area of residence.
† SE estimated by bootstrap with 1,000 replications.
‡ SE estimated according to Abadie and Imbens (2012), assuming that the conditional outcome variance is homoscedastic, i.e. does not vary with the covariates or treatment. This is implemented in Stata with the option `vce(iid)`. This assumption can be relaxed using the option `vce(robust, nn(2))` for the 1 match analysis and `vce(robust, nn(4))` for the 3 matches analysis.

Table 4: Standardised raw and weighted mean differences across exposure groups in the predictors used in the PS model for A_2; Simulation Learner PROBITsim Study: N=17,044

	Standardized differences	
	Raw	Weighted
Location= 2	-0.0034	0.0053
Location= 3	-0.0052	-0.0040
Location= 4	-0.0007	-0.0042
Mother's age (y)	0.2031	0.0084
Mother's age squared (y^2)	0.0696	0.0009
Educ= medium	0.0780	0.0033
Educ= high	0.1490	0.0028
Maternal smoking	-0.2945	-0.0061
Allergy	0.0026	-0.0006

Figure 1: Overlap in PS for A_2

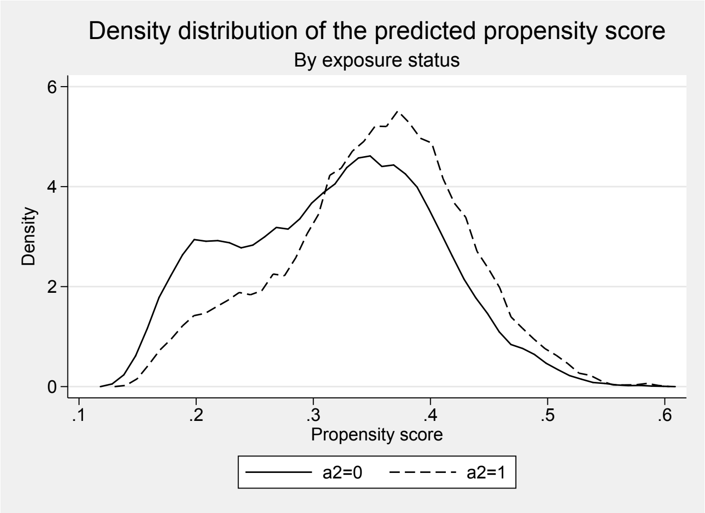

Table 5: Estimated ATE and ATT of A_3 on weight at 3 months (in grams) obtained using alternative estimation methods controlling for relevant confounders* and stratified by whether mothers were offered the BEP programme; PROBITsim Study.

		$A_1 = 0$		$A_1 = 1$	
Estimand	Estimation method	Estimate	(SE)	Estimate	(SE)
ATE					
	True value	386.8		422.3	
	Crude regression	503.2	(11.6)	582.0	(12.2)
	Regression adjustment (without interactions)	384.3	(2.8)	428.0	(3.3)
	Regression adjustment (with interactions)	384.7	(3.2)	425.3	(2.7)
	Regression with PS †	384.4	(3.2)	425.9	(3.3)
	PS stratification† (6 strata)	392.2	(4.1)	442.0	(6.5)
	PS matching (1 match)‡	386.5	(13.7)	429.0	(17.4)
	PS matching (3 matches)‡	380.7	(12.4)	437.2	(15.2)
	PS IPW†	384.7	(3.8)	426.6	(7.1)
	PS DR IPW†	384.8	(4.0)	426.7	(7.3)
ATT					
	True value	380.1		421.4	
	Regression adjustment (with interactions)	378.0	(2.9)	421.7	(2.5)
	PS stratification† (6 strata)	388.8	(4.8)	438.3	(9.5)
	PS matching (1 match)‡	384.3	(15.8)	435.6	(21.2)
	PS matching (3 matches)‡	387.9	(13.5)	441.2	(18.0)
	PS IPW†	381.9	(5.3)	429.2	(10.1)

* The variables controlled for in each of these analyses were: maternal age (linear and quadratic term), maternal education, maternal allergy status, smoking status in the first trimester (i.e. before programme allocation), area of residence, baby's birth weight (linear and quadratic term), whether birth was by caesarian section and, in the analyses restricted to $A_1 = 1$, whether the mother attended the programme.

† SE estimated by bootstrap with 1,000 replications.

‡ SE estimated according to Abadie and Imbens (2012), assuming that the conditional outcome variance is homoskedastic, i.e. does not vary with the covariates or treatment. This is implemented in Stata with the option `vce(iid)`. This assumption can be relaxed using the option `vce(robust, nn(2))` for the 1 match analysis and `vce(robust, nn(4))` for the 3 matches analysis.

Figure 2: Balance plot before and after matching on the PS for A_2

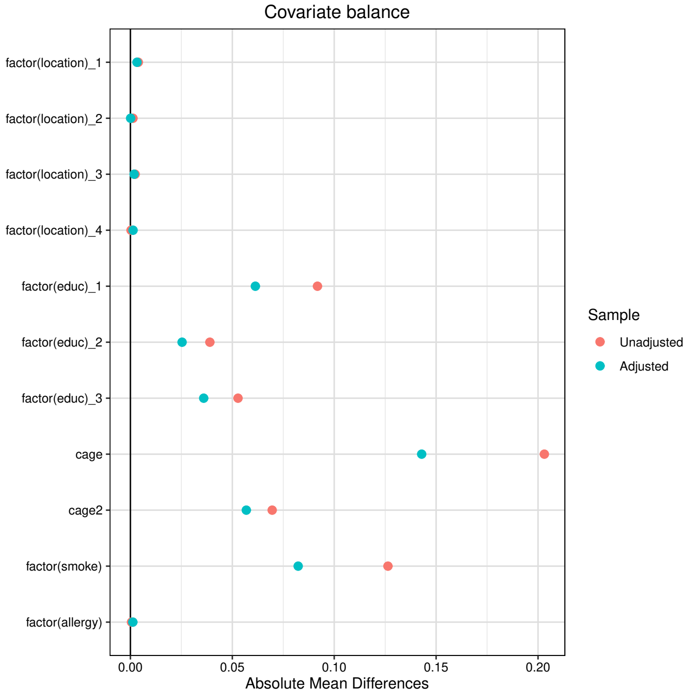

Figure 3: Overlap in PS for A_3 in the subset with $A_1 = 0$

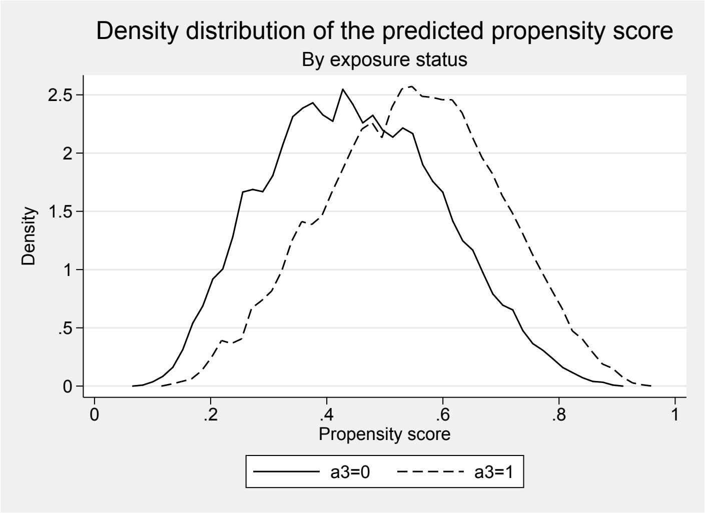

Table 6: Standardised raw and weighted mean differences across exposure groups in the predictors used in the PS model for A_3, separately by strata defined by A_1; Simulation Learner PROBITsim Study: N=17,044

	$A_1 = 0$		$A_1 = 1$	
	Standardized differences		Standardized differences	
	Raw	Weighted	Raw	Weighted
Location= 2	0.0416	-0.0053	-0.0083	.00035
Location= 3	-0.0227	-0.0029	0.0411	0.0242
Location= 4	-0.0234	0.0079	-0.0870	-0.0132
Mother's age (y)	0.4704	0.0044	0.5274	0.0025
Mother's age squared (y)	0.1358	0.0003	0.1423	-0.0010
Educ= medium	0.0344	0.0063	0.1246	0.0060
Educ= high	0.1847	0.0029	0.220	-0.0096
Maternal smoking	-0.3189	-0.0020	-0.4170	0.0027
Allergy	0.0074	-0.0094	0.0155	-0.0054
Caesarean birth	0.0294	0.0003	0.0250	0.0024
Male infant	0.0227	-0.0026	0.1131	0.0067
Birth weight (g)	0.3132	0.0052	0.3382	-0.0016
Birth weight squared (g^2)	0.0069	-0.0007	-0.0704	-0.0026

Figure 4: Overlap in PS for A_3 in the subset with $A_1 = 1$

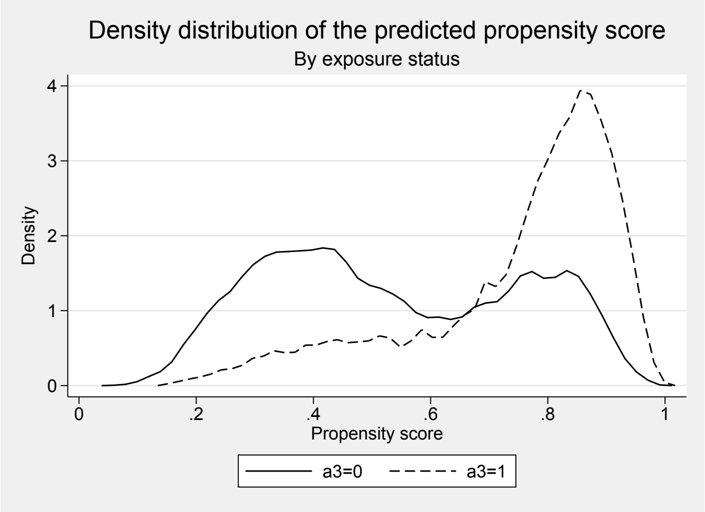

Figure 5: Balance plot before and after matching on the PS for A_3 in the subset with $A_1 = 0$

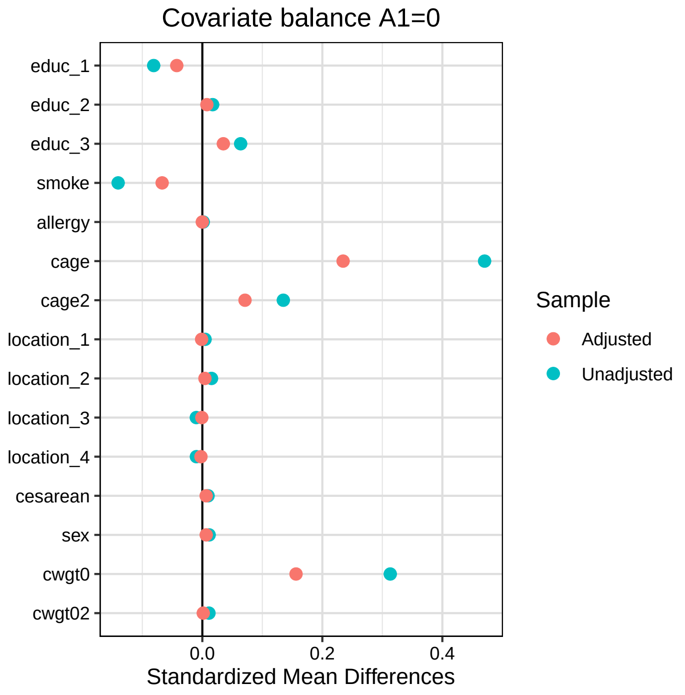

Figure 6: Balance plot before and after matching on the PS for A_3 in the subset with $A_1 = 1$

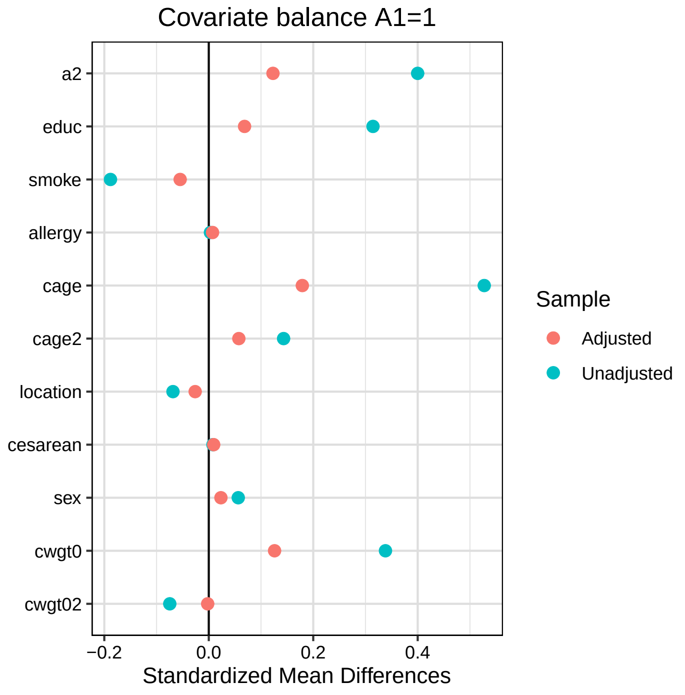

Table 7: Estimated ATE and ATT of A_3 on weight at 3 months (in grams) in the stratum of babies with low education mothers who were offered the BEP programme, obtained using alternative estimation methods controlling for relevant confounders*; PROBITsim Study.

Estimand	Estimation method	Estimate	(SE)
ATE			
	True value	476.7	
	Crude regression	626.1	(19.8)
	Regression adjustment (simple)	471.6	(6.5)
	Regression adjustment (with interactions)	481.0	(5.8)
	Regression with PS *	470.9	(6.5)
	PS stratification* (6 strata)	497.7	(10.7)
	PS matching (1 match)	482.3	(26.9)
	PS matching (3 matches)	472.6	(24.0)
	PS IPW	482.7	(11.0)
	PS DR IPW	482.7	(10.6)
ATT			
	True value	487.5	
	Regression adjustment (with interactions)	495.0	(5.2)
	PS stratification* (6 strata)	513.0	(16.1)
	PS matching (1 match)	510.1	(33.1)
	PS matching (3 matches)	489.7	(28.8)
	PS IPW	499.4	(17.2)

* The variables controlled for in each of these analyses were: maternal age (linear and quadratic term), maternal allergy status, smoking status in the first trimester (i.e. before programme allocation), area of residence, baby's birth weight (linear and quadratic term), whether birth was by caesarian section and, in the analyses restricted to $A_1 = 1$, whether the mother attended the programme.

† SE estimated by bootstrap with 1,000 replications.

‡ SE estimated according to Abadie and Imbens (2012), assuming that the conditional outcome variance is homoscedastic, i.e. does not vary with the covariates or treatment. This is implemented in Stata with the option `vce(iid)`. This assumption can be relaxed using the option `vce(robust, nn(2))` for the 1 match analysis and `vce(robust, nn(4))` for the 3 matches analysis.
